# Global Genetic Gain and Changes for Seed Yield, Agronomic, and Compositional Attributes in Soybean (*Glycine max* [L.] Merr.) from the 1920s to the 2010s: A Meta-Analysis

**DOI:** 10.3390/plants15172677

**Published:** 2026-08-31

**Authors:** Nhlakanipho Mbambo, Zamalotshwa Goodness Thungo, Alfred Odindo

**Affiliations:** School of Agriculture and Science, University of KwaZulu-Natal, Private Bag X01, Scottsville, Pietermaritzburg 3209, South Africa

**Keywords:** annual genetic change, biochemical attributes, crop improvement, micronutrient bioavailability, morpho-physiological traits, phytic acid, plant breeding, seed yield, seed oil content, seed yield, systematic review, yield-related attributes

## Abstract

The analysis of genetic gain and changes in economic traits among modern and older cultivars is important to estimate breeding progress, evaluate the effectiveness of selection methods, identify optimised and neglected attributes, detect trade-offs, and inform strategies in crop improvement programs. This meta-analysis analysed global genetic gain and changes in seed yield, yield-related traits, and seed compositional attributes in soybean (*Glycine max* [L.] Merr.). A meta-database comprising 272 genetic gain data points recorded from 29 studies and 2555 mean performances captured from 34 research papers was compiled from research reporting cultivars released globally between the 1920s and 2010s. The mean values extracted from these studies were subjected to the R-Software analysis of variance (ANOVA) to reveal the effects of year of cultivar release and production region on the studied agronomic and compositional attributes. Boxplots were constructed to visualise data distribution and variability, and Pearson correlation and principal component analysis (PCA) were performed to examine inter-relationships among the measured traits with year of cultivar release and production region. Significant differences (*p* < 0.001) in mean performance were found among decades of cultivar release for seed yield (SY), number of seeds per pod (NSPod), days to flowering (DTF), days to maturity (DTM), and total biomass (TB). Cultivars released in the 2000s attained the highest mean SY of 3737.62 kg ha^−1^, compared to 2156.08 kg ha^−1^ observed for varieties released in the 1960s. Annual mean increases for SY ranged from 1.75 to 47 kg ha^−1 ^year^−1^, driven primarily by improvements in number of seeds per plant (NSP), NSPod, plant height (PH), DTF, TB, and hundred seed weight (HSW). Conversely, seed protein content (SPC) declined across most breeding programs, while seed oil content (SOC) showed only modest improvement, and no consistent genetic gains were documented for micronutrient traits, including iron (Fe), zinc (Zn), calcium (Ca), or phytic acid (PA) content. Phytic acid, a potent chelator of essential minerals and a known inhibitor of protein digestibility in monogastric species, represents a critical but largely untracked compositional target in global soybean breeding. The absence of PA as a systematically monitored trait constitutes a significant gap in the literature, with direct implications for the nutritional quality of improved cultivars. Future breeding programs should adopt an integrated approach that simultaneously targets high SY, enhanced micronutrient bioavailability, and reduced antinutritional factor concentrations to advance both agricultural productivity and global food and nutrition security.

## 1. Introduction

The attainment of high genetic gain for economically important plant traits is a major focus in crop improvement programs of various cultivated crops. Genetic gain indicates annual percentage and actual changes in mean performance of desired plant traits due to artificial selection in breeding programs [1,2]. Positive or negative genetic gain can be recorded depending on the desired crop plant attribute. The assessment and documentation of genetic gain for economically important plant traits serve as a foundation for the formulation of demand-led breeding objectives and implementation of selection methods in plant breeding programs. Therefore, it is paramount to investigate the presence of positive and negative genetic gain or changes for economically important plant traits in agronomic crops such as soybean.

Soybean (*Glycine max* [L.] Merr.) is a world legume widely cultivated for its seed and foliage, which are important for human and animal consumption [3,4,5], and for biodiesel and vegetable oil production [6]. Soybean seed is rich in protein (i.e., 40%), with globulin making up 70% of the total seed protein, possessing well-balanced essential amino acids, including threonine, isoleucine, phenylalanine, leucine, lysine, methionine, valine, and tryptophan [7]. Other key macronutrients, including lipids, carbohydrates, and vitamins, are present in soybean seed [8]. Essential minerals such as iron, calcium, nitrogen, magnesium, potassium, and phosphorus are also present in soybean seed [9]. As a result, total global soybean production reached 388.098 million tonnes in 2021 [10], which was above expectations and attributed to high global demand for plant-based protein food resources by humans [11].

Despite the global importance of soybean as a nutrient-dense food resource for human consumption, its nutritional significance is compromised by the presence of phytochemical and antinutritional constituents in its mature seed, including phytic acid. Phytic acid is associated with reduced bioavailability of essential minerals such as iron, zinc, potassium, magnesium, and calcium in the human gastrointestinal tract [12]. Therefore, there is a requirement for soybean cultivars that possess enhanced genetic potential for key cultivation, yield, and nutritional characteristics, while maintaining low accumulation of phytochemical compositions to extend the legume crop’s role in global food and nutrition security.

An integration of classical and modern plant breeding approaches offers an opportunity to enhance genetic gain for key yield and compositional attributes in cultivated soybean cultivars and to improve the crop’s productivity. Breeders have mostly prioritised genetic improvement for increased seed yield, which has, in turn, affected genetic changes in other traits due to direct and indirect selection effects [13]. As a result, increases in genetic gain for agronomic yield-related traits such as enhanced number of branches (0.01 units year^−1^), number of pods (2.67 units year^−1^), total biomass (81.17 g m^−2^ year^−1^), and hundred seed weight (1.02 g year^−1^) have contributed to enhanced soybean productivity [14,15,16,17]. Other important agronomic traits (e.g., days to flowering, days to maturity, seed number per plant, pod length, seed width and thickness) and physiological characters (i.e., radiation use efficiency, harvest index, etc.) are important yield-influencing attributes in soybean, targeted by most global breeding programs [18,19,20,21]. Annual genetic increases ranging between 14 and 44 kg ha^−1^ year^−1^ exist for seed yield, reported in major soybean-producing regions, including Brazil, the USA, Argentina, and China [22,23,24,25]. Genetic improvement for enhanced nutritional attributes (i.e., amino acids, vitamins and isoflavones) and for low phytochemical (phytic acid and protease inhibitors) contents is a key objective in soybean breeding [26,27]. Nevertheless, there is limited breeding research reporting genetic gain and changes for most seed compositional attributes in soybean. These findings signify the need for a strategic approach to achieve simultaneous positive genetic changes for enhanced seed yield and its components, and for compositional attributes in soybean.

Modern and old cultivars of soybean possessing enhanced seed yield and related traits, and compositional attributes are [13,28,29] used for production and as breeding material in improvement programs. These cultivars share unstable performance under varied cultivation conditions [30,31,32,33], affecting the response to selection in breeding programs targeting to increase genetic gain for traits of interest. For example, in Iowa, improved soybean cultivars released between 1951 and 2004 were identified with variable seed yield performance in De Witt (3328 kg/ha) and Whiting (4733 kg/ha) [34]; similar outcomes were observed in cultivars released in Brazil and Argentina [25], displaying varying performance under different production regions. Molecular breeding using biotechnology tools, such as genotyping by sequencing and gene editing, to identify suitable genotypes based on quantitative trait loci (QTLs) instead of plant phenotypes has enhanced breeding progress in soybean [35,36,37]. Therefore, an evaluation of periodic and global genetic gain and annual change for yield and compositional attributes, associated with modern and older varieties, is important to inform strategies in current breeding programs [17]. A meta-analysis to provide scrutiny of global genetic gain and annual change for seed yield and its components, and for compositional attributes, is key to enhancing soybean breeding. It was hypothesised that genetic gain in seed yield as well as agronomic and compositional attributes of soybean differed significantly across breeding eras and production regions, with the new cultivars and those from major soybean-producing countries exhibiting higher rates of yield improvement. Therefore, the objective of this review is to provide an analysis of global breeding progress for seed yield and components, and for compositional attributes in soybean.

## 2. Results

### 2.1. Mean Genetic Gain and Changes for Agronomic Yield-Related Traits

Genetic gain and annual change values for agronomic attributes (i.e., yield and related traits) in global soybean breeding are presented in Table 1. Countries such as Zimbabwe recorded annual increases in mean number of days to flowering (DTF) for genotypes bred from 1966 to 2013, ranging from 0.15 (0.32% year^−1^) to 0.17 (0.35% year^−1^) days year^−1^, whereas Nigeria reported a decline in mean ranging from −0.12 (−0.25%) to −0.06 (−0.11%) days year^−1^ for the similar trait (Table 1). The genetic gain for days to maturity (DTM) ranged from −1.00 days year^−1^ to 1 day year^−1^ globally. The majority of the breeding regions identified a positive annual increase for DTM, except Brazil and the USA, which both revealed annual decreases. Regarding plant height, the majority of the breeding regions recorded annual decreases in mean PH, ranging between −0.01 and −2.1 cm year^−1^. Positive annual change in mean PH was reported in Zimbabwe, China, Nigeria, and the USA, recording increases of between 0.1 and 8.7 cm year^−1^. A highest genetic gain of 1.66% year^−1^ was recorded for increased plant height between 1980 and 1996 in Nigeria, followed by 0.5% year^−1^ recorded in Zimbabwe up to 2013. Lodging (LOD) recorded a majority of annual decreases across the globe, with values of between −0.012 and −2.4 units year^−1^ captured between 1923 and 2013.

The number of branches of soybean genotypes released in Brazil between the years 1965 and 2011 was found to decrease in genetic mean by −0.12 units year^−1^, while genotypes released between 1958 and 2001 in China showed an annual increase of 0.01 units year^−1^. The number of pods per plant (NPP) of cultivars released between 1969 and 1993 in India declined by −4.00% year^−1^, similar to the decline of −0.22 (−0.16%) units year^−1^ reported for cultivars released between 1980 and 1996 in Nigeria (Table 1). Interestingly, Nigeria further reported an increase of 2.67 (3.51%) in genetic gain for NPP for cultivars released between 1980 and 1996. Also, China documented a high annual increase of 1.8 and 2.1 units year^−1^ between 1995 and 2016. Regarding the number of seeds per pod (NSPod), India (10% year^−1^) and China (1.9 units year^−1^) have reported the highest positive genetic gain for NSPod cultivars released between 1969 and 1993 and 1995 and 2016, respectively. On the other hand, Brazil recorded a negative genetic gain of −0.45 (−1.1%) units year^−1^ for NSPod among cultivars released between 2000 and 2017. Canada reported the highest (0.49% year^−1^) positive genetic gain for number of seeds per plant (NSP) with cultivars released between 1934 and 1992, and a negative gain (−0.31 units year^−1^) was recorded in China for the same character among cultivars released between 1929 and 2004. The USA reported varying results with regard to genetic gain for hundred seed weight (HSW), as it recorded a decrease of −1.14 (7.7%) g year^−1^ and later recorded an increase of 1.02 (7.8%) g year^−1^ in HSW displayed by cultivars released between the years 1923 and 2008 (Table 1). Nigeria reported a decrease of −6.07 (−0.41%) g m^−2^ year^−1^ for total biomass (TB) by cultivars bred between 1980 and 1996, contrary to these results. Brazil reported an 81.17 g m^−2^ year^−1^ increase by cultivars bred between 1965 and 2011. Furthermore, India also observed a 5% year^−1^ increase by cultivars released between 1969 and 1993 (Table 1). For seed yield (SY), most global regions reported an increase in genetic gain for seed yield (SY), with Zimbabwean cultivars showing the highest annual mean increase of 47.00 (1.67%) Kg ha^−1^ year^−1^, which were released between 1966 and 2013. Indian cultivars released between 1969 and 1993 showed a 19% year^−1^ annual increase for SY. The positive genetic gain for SY ranged from 0.45% year^−1^ (Canada, 1934–1992) to 58.5% year^−1^ (USA, 1950–2000). Only the USA reported a decline of −1.4% year^−1^ in genetic gain for SY (Table 1).

### 2.2. Mean Genetic Gain and Changes for Physiological Yield-Related Traits

Genetic gain and annual change values for physiology-based attributes in global soybean breeding are presented in Table 1. The genetic mean for stomatal conductance in soybean increased by 0.002 molH_2_O m^−2^s^−1^ and 0.48% year^−1^ in Brazil and Canada, respectively. Moreover, Brazil reported an increase of 0.11 and 0.04 units year^−1^ mean for both chlorophyll a and b, respectively, shown in cultivars bred between 1965 and 2011. Both China and Canada observed a decline of 0.31 and −0.38% year^−1^ in genetic gain for leaf area index (LAI) between the years 1950 and 2006 and 1934 and 1992, respectively, whereas Brazil reported a 0.31% year^−1^ increase for the same trait displayed between the years 1965 and 2011. Water use efficiency (WUE) was reported to decrease on average by −0.002% year^−1^ in Brazil for cultivars released between 1965 and 2011. A 0.08 µmolCO_2_ m^−2^s^−1^ year^−1^ increase in mean photosynthetic rate was reported in Brazil, measured on cultivars bred between 1965 and 2011, and China also measured an increase of 0.59% year^−1^ in genetic gain for cultivars bred between 1950 and 2006 (Table 1). Additionally, cultivars bred between 1965 and 2011 in Brazil resulted in an increased mean of 0.04 mmol m^−2^s^−1^ in transpiration rate.

### 2.3. Mean Genetic Gain and Changes for Seed Compositional Traits

There was a decrease in genetic gain for seed protein content (SPC), with the USA showing the highest annual mean decrease of −0.35 g Kg^−1^ year^−1^ among cultivars bred between 1920 and 2000. Contrary to these findings on SPC, the USA again documented a high genetic mean increase of 0.18 g Kg^−1^ year^−1^, recorded among cultivars released between 1950 and 2000. Regarding seed oil content (SOC), most countries reported an increase in genetic mean, with the highest (0.142 g kg^−1^ year^−1^) reported by the USA among cultivars bred between 1923 and 2008. The second highest genetic mean increase in SOC (0.136 g Kg^−1^ year^−1^) was associated with soybean cultivars released between 1923 and 2008 in the USA.

### 2.4. Summary Statistics for Agronomic Traits, Yield-Related Traits, and Seed Quality Traits Among Soybean Genotypes from Diverse Production Regions

The mean values of traits DTF, DTM, PH, SY, NSPod, and TB differed significantly (*p* < 0.001) among soybean cultivars cultivated across diverse global production regions (Table 2). DTF ranged between 36.48 and 63.81 days, with an average of 48.57 days, recorded for Colombia and Bangladesh in that order. In terms of DTM, soybean cultivars grown in Zimbabwe took longer to mature (124.76 days) compared to those grown in the USA (51.50 days) and compared to the global average value of 103.89 days. The tallest (99.90 cm) and shortest (32.50 cm) were reported in Croatia and Vietnam, respectively, with a mean global PH of 71.42 cm. A mean value of 2.36 was recorded for NSPod, with the highest number of seeds per pod (3.10) reported in soybean cultivars grown in Ethiopia. Soybean cultivars grown in Bangladesh had fewer seeds (1.96) in comparison to all the production regions observed in the current study. The highest mean SY was observed in cultivars cultivated in Zimbabwe (4200 Kg/ha), whereas the lowest value (1657.24 Kg/ha) was observed in cultivars grown in Ethiopia. Also, the mean TB value was the highest (9344.01 Kg/ha) in cultivars grown in Ethiopia compared to those grown in Nigeria (1819.81 Kg/ha).

**Figure 1:**
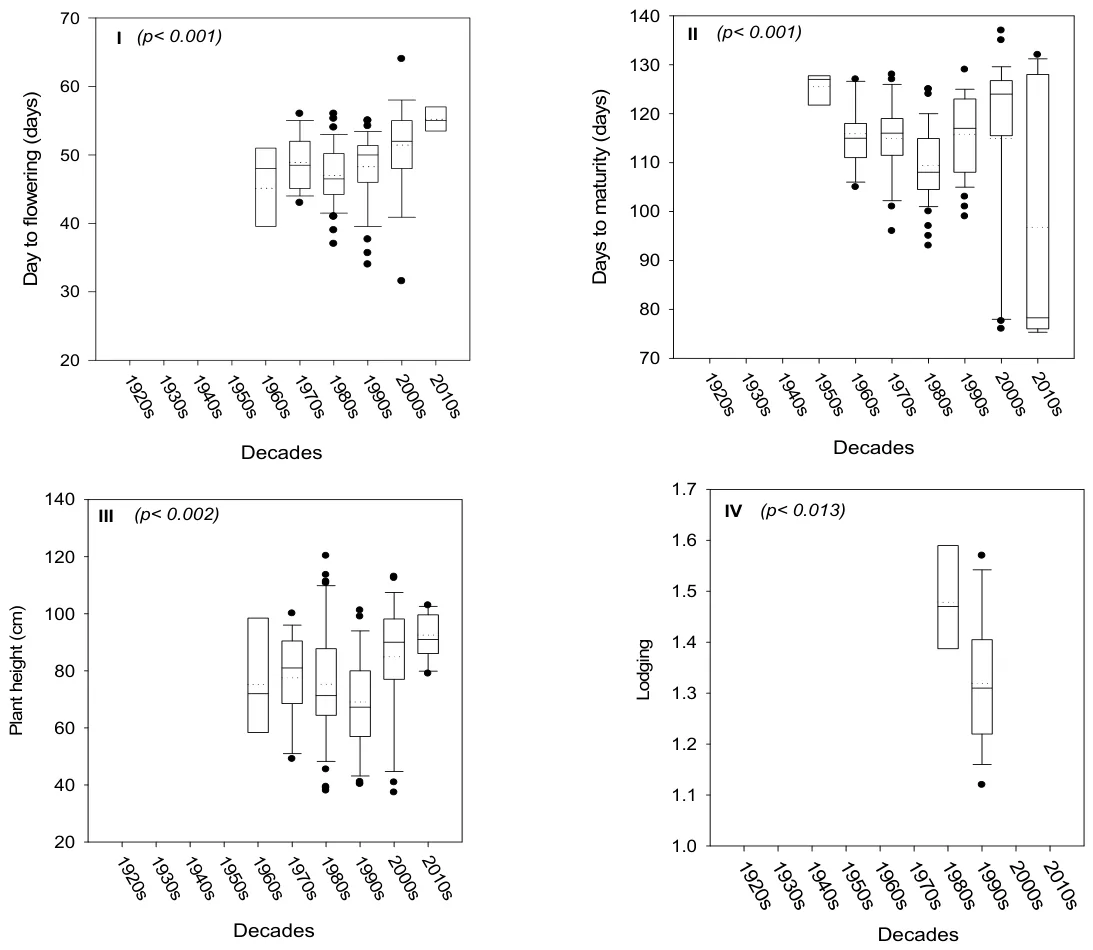
A variation in the means of agronomic traits reported for the different decades. (**I**)—days to flowering, (**II**)—days to maturity, (**III**)—plant height, and (**IV**)—lodging.

The mean values for DTF, DTM, PH, and LOD differed significantly (*p* < 0.05) among soybean cultivars cultivated across different decades of cultivar release (Figure 1). Mean DTF differed significantly (*p* < 0.001) across the different decades of cultivar release in soybean. As a result, soybean cultivars released in the 2010s showed a high number of days to flowering (55.4 days) compared to those released in the 1960s (45.2 days). For DTM, genotypes released in the 1950s matured late, recording high DTM (125.50 days) compared to those released in the 2010s (97.20 days). Soybean cultivars released in the 1990s were, on average, shorter (69.29 cm) compared to those released in the 2010s (93.80 cm). LOD mean values were higher for cultivars released in the 1980s (1.48 units) compared to varieties released in the 1990s (1.32 units).

**Figure 2:**
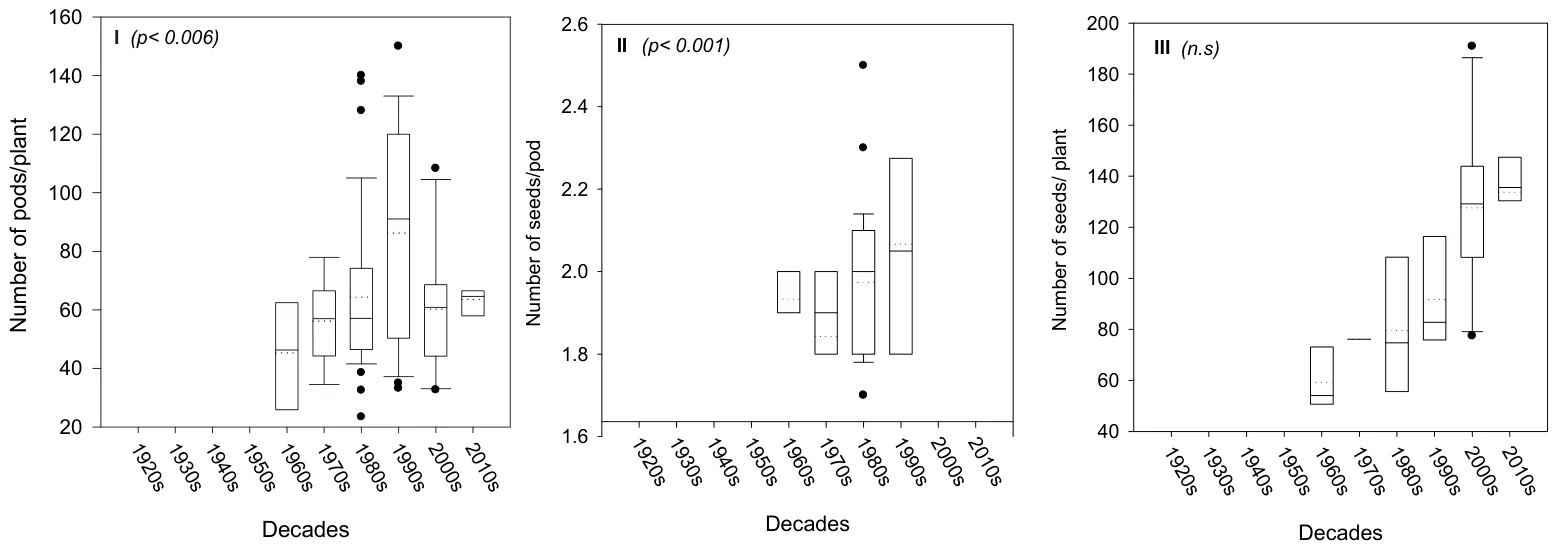
A variation in the means of agronomic traits reported for the different decades. (**I**)—number of pods/plants, (**II**)—number of seeds/pod, and (**III**)—number of seeds/plants.

**Figure 3:**
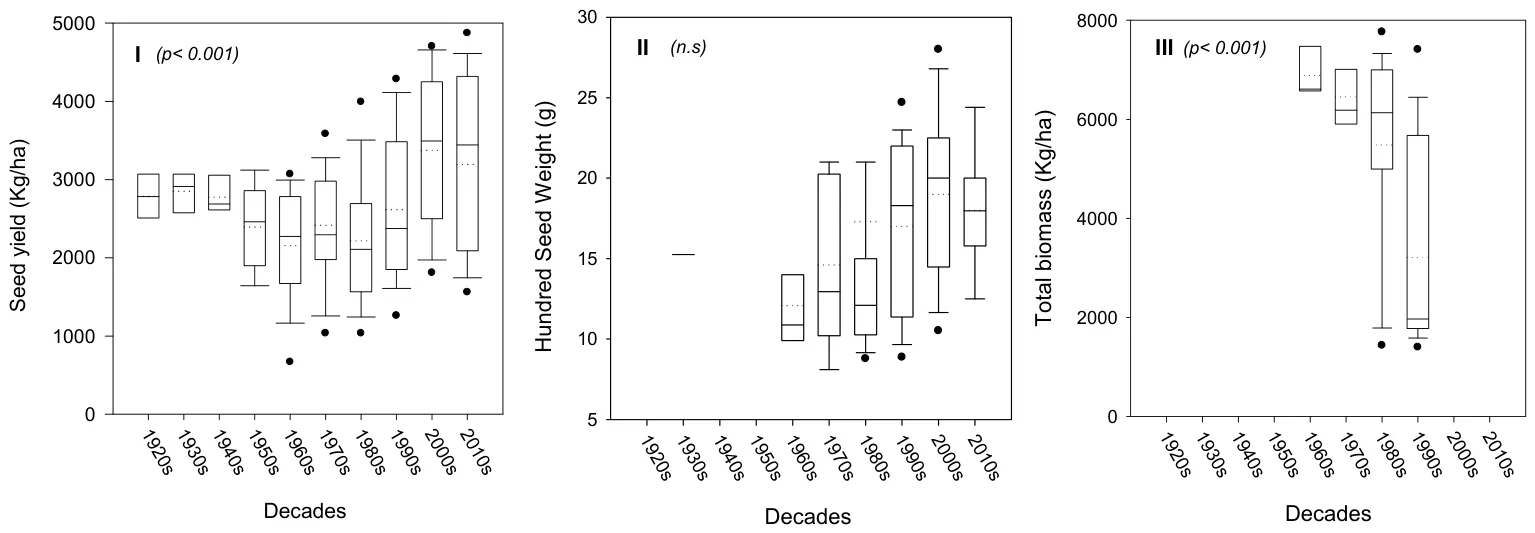
A variation in the means of agronomic traits reported for the different decades. (**I**)—seed yield, (**II**)—hundred seed weight, and (**III**)—total biomass.

**Figure 4:**
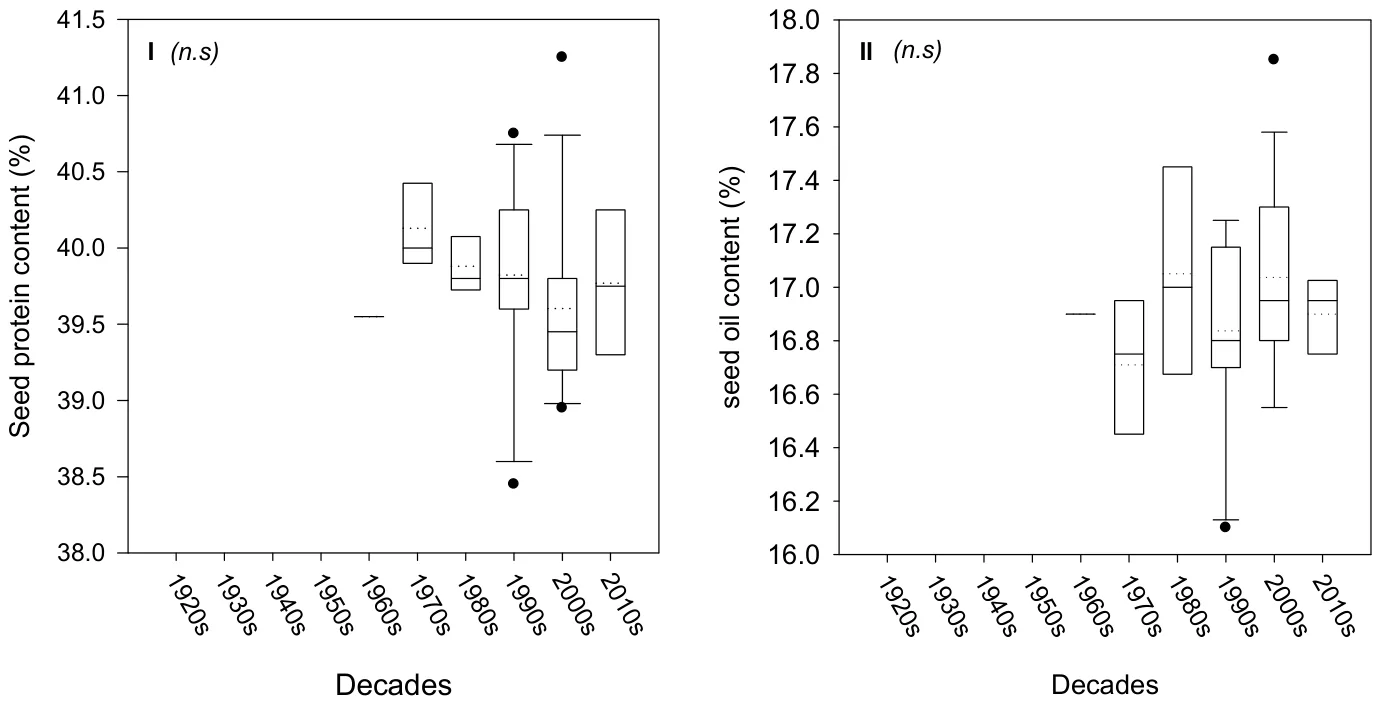
A variation in the means of biochemical traits reported for the different decades. (**I**)—seed protein content and (**II**)—seed oil content.

The mean values for NPP and NSPod differed significantly (*p* < 0.05) among soybean cultivars cultivated across different decades of cultivar release (Figure 2). Soybean cultivars released in the 1990s produced more NPP (86.80 units) compared to those released in the 1960s (45.22 units). Regarding NSPod, cultivars released in the 1990s showed the highest mean of 2.07 units, whereas those released in the 1970s recorded the lowest mean (1.84 units). The mean values for SY and TB differed significantly (*p* < 0.05) among soybean cultivars cultivated across different decades of cultivar release (Figure 3). For SY, cultivars released in the 2000s showed better performance (3373.62 Kg ha^−1^) compared to those released in the 1960s (2156.08 Kg ha^−1^). Regarding TB, soybean cultivars released in the 1960s produced the highest mean (6885.00 Kg ha^−1^) compared to those released in the 1990s (3210.16 Kg ha^−1^). The seed compositional attributes showed no significant difference across the breeding decades (Figure 4).

### 2.5. The Association Between the Agronomic and Biochemical Traits Reported for the Different Release Decades and Countries

Bivariate correlation coefficients and significance tests for the agronomic and biochemical soybean traits recorded across decades and diverse global production regions are presented in Table 3. SY showed high positive and significant (*p* ≤ 0.05) correlations with DTF (*r* = 0.504), PH (*r* = 0.45), NSPod (*r* = 0.84), NSP (*r* = 0.657), TB (*r* = 0.263), HSW (*r* = 0.255), and SOC (*r* = 0.40). Conversely, a negative and significant (*p* < 0.001) correlation was recorded between SY and SPC (*r* = −0.49, which was also negatively correlated with SOC (*r* = −0.609, *p* < 0.001) and HSW (*r* = −0.431, *p* < 0.01). Correlation analysis also showed a positive and significant association between SOC and HSW (*r* = 0.31, *p* < 0.05). DTF was positively and significantly (*p* ≤ 0.01) correlated with DTM (*r* = 0.829), PH (*r* = 0.55), and NPP (*r* = 0.58), whereas negative associations were revealed with NSPod (*r* = −0.39) and TB (*r* = −0.47). More other positive and significant correlations were recorded, including NPP with NSPod (*r* = 0.44, *p* < 0.001) and NSP (*r* = 0.90; *p* ≤ 0.001).

**Figure 5:**
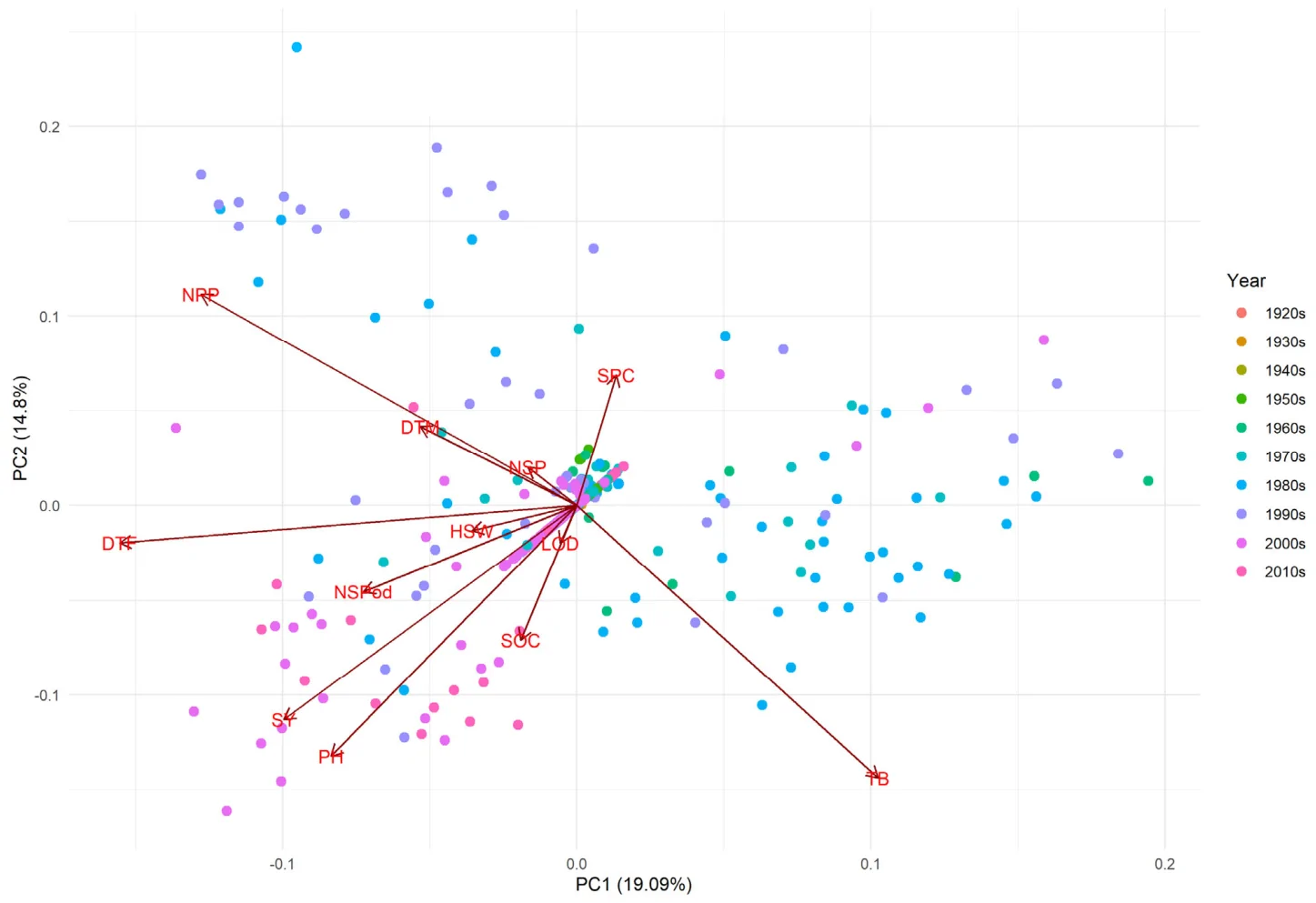
Principal component analysis (PCA) biplot of soybean traits of cultivars released across different decades (ranging from the 1920s to the 2010s). The vectors (denoted with red arrows) show the direction and magnitude of specific soybean traits contributing to the observed variation and their correlations with one another. Where DTF—number of days to flower (in days), DTM—number of days to maturity (in days), PH—plant height (in cm), LOD—lodging, NPP—number of pods per plant (number), NSPod—number of seeds per pod (number), NSP—number of seeds per plant (number), SY—seed yield (Kg/ha), HSW—hundred seed weight (g), TB—total biomass (Kg/ha), SPC—seed protein content (%), SOC—seed oil content (%).

**Figure 6:**
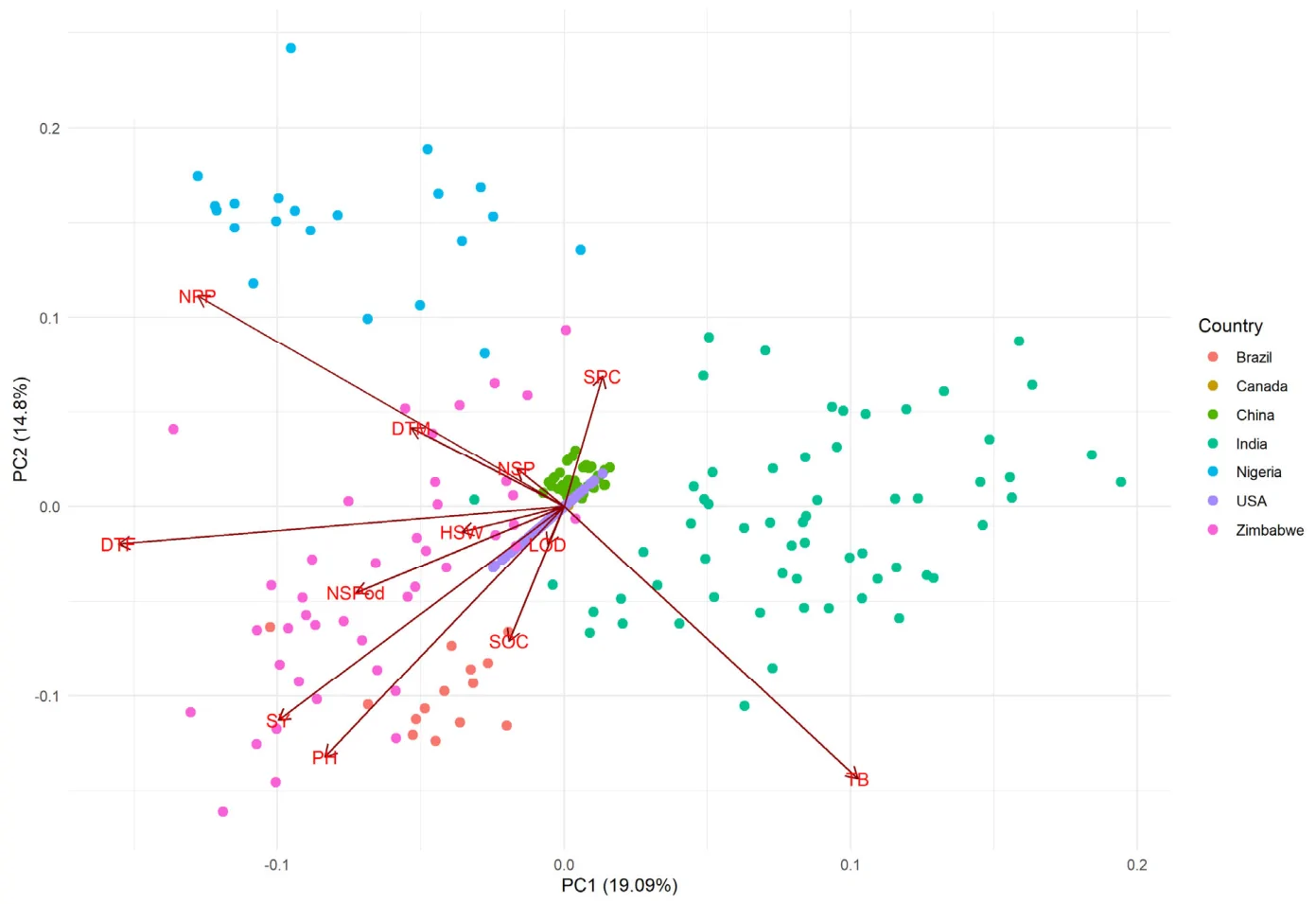
Principal component analysis (PCA) biplot of soybean traits of cultivars grown across different countries (Brazil, Canada, China, India, Nigeria, USA, and Zimbabwe). The vectors (denoted with red arrows) show the direction and magnitude of specific soybean traits contributing to the observed variation and their correlations with one another. Where DTF—number of days to flower (in days), DTM—number of days to maturity (in days), PH—plant height (in cm), LOD—lodging, NPP—number of pods per plant (number), NSPod—number of seeds per pod (number), NSP—number of seeds per plant (number), SY—seed yield (Kg/ha), HSW—hundred seed weight (g), TB—total biomass (Kg/ha), SPC—seed protein content (%), SOC—seed oil content (%). USA—United States of America.

Principal component biplots showing the interrelationship of traits with year of release and production region are presented in Figure 5 and Figure 6, respectively. The PCA biplot based on PC1 (19.09% variance) and PC2 (14.8% variance) showed a clear temporal transition in trait associations across decades of cultivar release (Figure 5). From the 1960s to the 2010s, soybean cultivars showed high stability for increased SPC. TB was positively associated with soybean cultivars released in the 1980s. The majority of modern soybean cultivars released in the 2000s and 2010s were associated with reduced PH, SY, NSPod, HSW, and SOC. Nevertheless, the 2000s and 2010s were associated with reduced LOD. Regarding the interrelationship between recorded soybean attributes and production region (Figure 6), cultivars released in India and China showed a positive association with enhanced SPC and TB in soybean. Soybean cultivars in Brazil and Zimbabwe were mostly associated with reduced PH, SY, NSPod, HSW, and SOC.

## 3. Discussion

### 3.1. Global Genetic Changes for Seed Yield and Related Attributes over Decades of Soybean Breeding

On average, most of the studies included in the current review have reported an increase in genetic gain for seed yield (SY) of soybean. The most significant increases were reported by Brazil, Argentina, and Zimbabwe with genetic means of 40.06 (2.4%), 44.3 (1.1%), and 47.00 (1.67%) Kg ha^−1^ year^−1^, respectively, by cultivars bred between 1965 and 2015 [17,38,40]. These gains may reflect a significant modernisation of the crop’s genetic potential in these regions. The increases in yield genetic gains reported in Brazil, particularly, are important as they meet a 2.4% year^−1^ annual growth rate required to double global food production by 2050 [59]. However, this comparison is only an approximate benchmark, as linear genetic gain and compound production growth are methodologically different, and achieving this genetic potential in the field will also depend on agronomic and environmental factors. Moreover, when comparing cultivars released in the 1960s to those of the 2000s, there has been a ~73.35% increase in the past four decades. The commercial introduction of the Roundup Ready (RR) soybean cultivars in the 1990s, which were genetically modified to be resistant to the herbicide glyphosate, could be another reason for improved genetic gains in yield [60]. In Brazil, yields changed due to the expansion of production land, introducing new cultivars, and transgenic cultivars, which exhibited an indeterminate growth habit and were able to adapt to no tillage management practices [61]. Lopez et al. [19] also mentioned that there has been an increase in soybean yields in the last century because of improved cultivars and better agronomic practices.

Similar to SY, other yield components also showed significant global genetic gain changes. In regions of India, Brazil, Nigeria, the USA, and China, there have been reports of an increased genetic mean for number of pods per plant (NPP) and number of seeds per pod (NSPod) ranging from 0.11 to 2.67 units year^−1^ and 0.002 to 1.9 units year^−1^ by cultivars released between 1965 and 2017 and 1923 and 2016, in that order [14,29,43,46,50]. This could suggest that modern breeding has modified plant architecture, resulting in more efficient reproductive growth to yield a high number of pods and seeds per plant in recently released cultivars. Interestingly, trends showed no significant changes in hundred seed weight (HSW); this could mean that this trait has not been a major interest in breeding programs. And this could suggest that yield is not largely driven by seed size but more by seed numbers. Contrary to the synthesis of this study, a number of studies have reported that the new cultivars have higher HSW compared to the older ones [23,62].

The trends suggested a decline in genetic gains in total biomass (TB), comparing the genetic means of older cultivars released in the 1960s with those released in the 1990s. This implies that recent cultivars have improved resource allocations, resulting in higher seed yields compared to the stover. Conversely, in Nigeria, cultivars released between 1980 and 1996 showed contrasting results, and it was reported that TB decreased by −6.07 (−0.41%) g m^−2^ year^−1^, and the authors also noticed a 16.18 (1.31%) g m^−2^ year^−1^ [14]. Increases in genetic gain for number of days to flowering (DTF) and maturity (DTM) have been seen among modern cultivars released around the 2010s compared to those released between the 1950s and 1960s. These findings signify that breeding programs have been focusing on selecting for late flowering and early maturing cultivars. The delay in flowering in modern cultivars implies that the pre-reproductive stage is extended, subsequently maximising biomass accumulation. Also, the shortening of the maturation period reflects the breeder’s deliberate selection for short-season lines, which facilitates double cropping systems. Flowering time is an economically important trait in soybean which affects yield performance. For instance, Fudge [63] reported that early-flowering plants produced lower yield compared to those that flowered later. Regarding DTM, some studies have reported varying results where the cultivars released in the same years showed both an increase and a decrease in genetic mean. Tagliapietra et al. [64] highlighted that the maturation period of soybean is also influenced by the response of the cultivars to photoperiod, which is affected by the latitude of the production environment, and that DTM is used to classify cultivars. Therefore, genotype x environment interaction (GEI) becomes an important factor to consider for DTM improvement in soybean [32,65].

Regarding plant height (PH), the newer cultivars released in the 2010s seem to be taller compared to those released in the 1990s, as the genetic gain in plant height (PH) was found to be ~35.37%. Similarly, an increase in genetic gain (0.11–0.5% year^−1^) for PH was reported in Zimbabwe for cultivars released between 1966 and 2013 [38]. These signify that breeding has managed to alter the morphological structure of modern soybean cultivars in the past two decades. However, numerous studies have reported a decrease in genetic mean for PH ranging from −2.1 to −0.01 cm year^−1^ among cultivars released between 1920 and 2017 [29,39,47]. Umburanas et al. [23] reported a negative relationship between PH and the year of release in soybean cultivars. Rogers et al. [39] reported that in the USA, breeders have been opting for shorter soybean varieties that are associated with increased seed yield per area, whereas taller varieties are prone to lodging (LOD) and produce few pods per plant. Regarding LOD, the results indicated that the cultivars released around the 1990s are able to maintain their upright architecture compared to those released in the 1980s. The trends also show that there has been progress made by breeders in developing cultivars that are less prone to lodging, as plenty of studies reported a decrease in genetic mean for LOD, ranging from −2.4 to −0.012 units year^−1^ by cultivars released between 1923 and 2013 [13,14,17,23,38]. In contrast to these results, other studies have observed an increase in genetic mean for LOD ranging from 1.8 to 2.4 units year^−1^ by cultivars released between 1923 and 2008 [49]. Cultivars with more upright canopies have been found to have high photosynthetic efficiency, especially during pod filling, and are associated with enhanced yield [23,56]. Rincker et al. [13] also mentioned that reduction in LOD is among the most important plant attributes considered in soybean cultivar design and development.

Regarding physiology-based attributes, genetic changes are less frequently documented compared to morphology-based traits. Nevertheless, modern breeding has managed to improve stomatal conductance (0.48% year^−1^), chlorophyll content a and b (0.11 and 0.04 units year^−1^, respectively), and photosynthetic (0.59% year^−1^) and transpiration rate (0.04 mmol m^−2^ s^−1^ year^−1^) amongst the new cultivars [17,48,57,63]. In contrast, negative genetic gain for leaf area index (−0.38% year^−1^) and water use efficiency (−0.002% year^−1^) have also been reported by other studies [17,57]. The limited report of genetic gain and annual mean changes for physiology-based traits suggests the need to include their enhancement as key breeding objectives in current improvement programs.

### 3.2. Global Genetic Gains for Seed Compositional Attributes over Decades of Soybean Breeding

The seed compositional quality traits of soybean cultivars have shown no significant genetic mean changes, with no substantial genetic gains in both seed protein content (SPC) and seed oil content (SOC) among modern cultivars. These findings may imply that selection in breeding programs has been aimed at high yield and its related traits. Also, Umburanas et al. [23] reported that there was a negative and positive correlation between SPC and SOC with cultivar year of release, in that order. In addition, studies have indicated that there has been a decrease in genetic progress for seed protein content ranging from −0.018 to −0.01% year^−1^ by cultivars released between 1920 and 2015 from countries like Zimbabwe, the USA, and Argentina [13,38,40,49,52]. Compared to SPC, studies have reported positive genetic gain for seed oil content ranging from 0.0062 to 0.142% year^−1^ in cultivars bred between 1920 and 2013 from countries like Zimbabwe and the USA [13,39,41,54]. Despite numerous studies reporting genetic gain in SPC and SOC, the documentation of genetic gain in other compositional traits such as iron (Fe), zinc (Zn), calcium (Ca), and phytic acid (PA) remains limited [26,66]. PA is of economic importance due to its ability to chelate essential micronutrients present in soybeans, thereby reducing their bioavailability [67,68]. Furthermore, as an antinutrient, PA forms indigestible complexes with proteins, meaning that even when SPC is high, the protein may not be bioavailable to monogastric animals or humans who lack the endogenous phytase enzyme required to degrade these complexes [69,70]. This gap in the literature may have a dire effect on breeding programs because high-yielding modern cultivars might have been developed; however, they have elevated PA concentrations, which subsequently reduces the bioavailability of essential micronutrients such as Fe, Zn, and Ca. This, therefore, signifies the need to incorporate breeding objectives that are related to enhancement of key seed compositional attributes in current soybean breeding programs.

### 3.3. Traits Association and Their Strategic Implications for Future Breeding

Understanding the correlation between soybean yield and its components, and with compositional attributes, is important for informing and refining selection strategies and breeding objectives in breeding programs. Also, understanding trait associations could provide avoidance of genetic trade-offs between economically important soybean traits, which limits the efficiency of modern soybean cultivars [21,71]. In the present review of the literature, increases in SY were closely associated with high DTF, PH, NSPod, NSP, TB, HSW, and SOC, represented by positive and significant correlations between SY and these component traits. Therefore, this makes these traits valuable for multivariate selection strategies with the aim of developing high-yielding cultivars. For instance, China increased SY by increasing the HSW and number of seeds per plant (NSP) [72]. Moreover, the principal component analysis (PCA) suggested that there is a temporal evolution in soybean breeding, with the newer cultivars released from the 2000s to the 2010s exhibiting strong association between genetic changes made in SY, PH, SOC, and NSPod. Other studies have also reported positive correlations between SY and physiological traits, such as chlorophyll content, photosynthetic rate, and transpiration rate, supporting indirect selection for enhanced SY through these traits [17]. The inverse relationship between SY and SPC is undesirable because protein is one of the most important compositional quality attributes in soybean. The improvement of soybean yield has been at the expense of SPC, negatively affecting the multiple uses of soybean [23]. On the other hand, SOC is strongly correlated with SY and negatively associated with SPC. Both SPC and SOC compete for the limited photo-assimilates during seed development, and that is the primary reason they are negatively associated with each other [73]. Therefore, this makes it a cumbersome task to develop cultivars that are high in SPC and SOC and are high yielding. Current soybean breeding programs could target systematic stacking of genes to develop novel cultivars possessing combined high SPC and SOC.

A methodological limitation of this meta-analysis is that the raw yield and genetic gain data were combined and statistically compared across production systems that differ substantially in socioeconomic development, technological input, and macroclimatic conditions. Pooling data from high-input, highly mechanised, and often irrigated systems with low-input systems within a single global analysis may confound the true genetic signal for breeding progress with underlying environmental and management variability. This limitation should be considered when interpreting the reported global trends, and future meta-analyses with larger, more geographically balanced datasets are encouraged to adopt a stratified approach to disentangle genetic and environmental sources of variation in soybean genetic gain. Moreover, since the dataset includes both registered commercial cultivars and experimental breeding lines, particularly for some developing regions, the estimated genetic gain may not fully reflect gains realised in commercial, on-farm production. Experimental lines are typically evaluated under more controlled trial conditions, which could bias estimates of realised genetic gain. This distinction should be considered when extrapolating these findings to global commercial agriculture.

## 4. Materials and Methods

### 4.1. Study Setup and Data Collection

**Figure 7:**
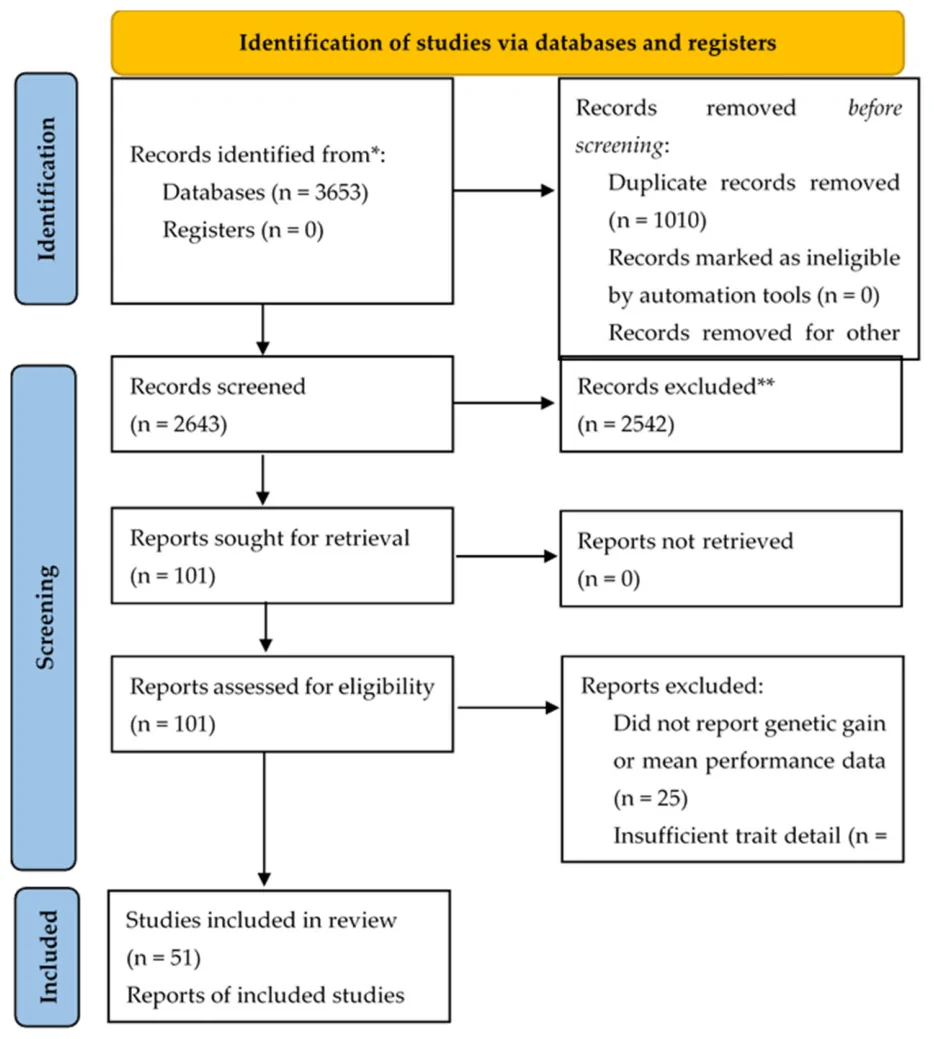
PRISMA 2020 flow diagram illustrating the identification, screening, and inclusion of studies for the meta-analysis of soybean genetic gain and mean performance [74]. A total of 3653 records were identified through database searches, of which 51 studies met the eligibility criteria and were included in the final synthesis. * Consider, if feasible to do so, reporting the number of records identified from each database or register searched (rather than the total number across all databases/registers). ** If automation tools were used, indicate how many records were excluded by a human and how many were excluded by automation tools.

The meta-analysis was conducted using metadata collected from published scientific research papers reporting the response of seed yield and its components, and of seed compositional attributes in soybean. The research papers were accessed from academic search engines such as Google Scholar, Science Direct, Springer Link, Scopus, and Web of Science. The following search words were used to find relevant research papers: “genetic gain”, “genetic changes”, “soybean”, “grain yield”, “yield-related traits”, and “compositional traits”. The following criteria were considered in the identification of research papers: (i) the paper needed to report annual genetic gain and changes, mean values on seed yield and related traits, and compositional attributes in soybean; (ii) the papers were selected regardless of test region or environmental, production, or test conditions; and (iii) the papers were selected regardless of year of study. Two databases were created for analyses: the first database recorded annual genetic gains and changes for seed yield and related traits and for compositional attributes. The second database captured mean performance values for seed yield and related traits, and for compositional attributes. These databases recorded articles and study details (i.e., author names, year of publication, country where the study was conducted), and soybean germplasm information (i.e., cultivar name and year of release). The compiled database includes both officially registered commercial cultivars and experimental breeding lines or germplasm accessions evaluated in multi-environment trials. Where studies did not explicitly distinguish between commercial and experimental genotypes, entries were retained as reported in the original source and are collectively referred to as “cultivar”. The first database consisted of 272 data points retrieved from 29 research papers. The second database comprised 2555 data points derived from 34 research papers. The two databases shared 12 papers in common, resulting in a total of 51 studies included in the review (Figure 7). The details of research papers included in the compilation of the genetic gain and changes database are presented in Table 4. Information on research papers constituting the database of mean performances for seed yield and related traits and compositional attributes is shown in Supplementary Table S1.

### 4.2. Definition of Seed Yield and Related Traits, and Compositional Attributes Reviewed

The description of seed yield and related traits, and compositional attributes included in the current analysis, is presented in Table 5. Seed yield-related traits included agronomic attributes such as days to 50% flowering, days to 50% maturity, plant height, lodging, number of branches per plant, number of pods per plant, number of seeds per pod, number of seeds per plant, total biomass, and hundred seed weight. Other yield-related traits included physiological attributes such as stomatal conductance, chlorophyll content (a and b), leaf area index, water use efficiency, and photosynthetic and transpiration rates. The compositional attributes included seed protein and oil content.

### 4.3. Data Analysis

The years in which the cultivars were released were grouped based on decade, i.e., cultivars released in 1923 were grouped as 1920s (1920–1929), whereas the 1930s included those released between 1930 and 1939, 1940s (1940–1949), 1950s (1950–1959), 1960s (1960–1969), 1970s (1970–1979), 1980s (1980–1989), 1990s (1990–1999), 2000s (2000–2009), and 2010s (2010–2019). The data distribution of the traits was summarised using boxplots, which showed the minimum, maximum, median, mean (denoted in dotted lines), the first and third quartiles, and the outliers. The boxplots (Figure 1, Figure 2, Figure 3 and Figure 4) were constructed using SigmaPlot software—version 10.0 [75], and Figure 5 boxplots were created using RStudio software version 4.5.3 [76]. Varieties for which the year of introduction was not documented in the source literature (Table S1) were excluded from the decade-by-decade analyses, as this classification required a known release year. In addition, there are traits and countries that were not included in the analysis because data from journal articles were limited. The mean values were subjected to an unbalanced analysis of variance (ANOVA) on Genstat 23rd edition [77]. The means of the traits between the decades, countries, and their interaction were separated using the least significant difference (LSD) at a 0.05 significance level. Pearson’s correlations, principal component analysis (PCA), and PCA biplots were derived using RStudio software version 4.5.3 [76].

## 5. Conclusions

This review has demonstrated that global soybean breeding programs have achieved consistent genetic gains in yield and related traits over the past several decades. Modern soybean cultivars released from the 1990s to 2010s showed improved seed yield (SY), days to flowering (DTF), and plant height (PH) of ~73.35%, 22.3%, and 35.37%, respectively, compared to varieties released between the 1950s and 1960s. This was mainly displayed by cultivars released in countries like Brazil, Zimbabwe, and India. These improvements in yield are driven largely by improvements in yield components such as number of seeds per plant (NSP), number of seeds per pod (NSPod), and optimised flowering. However, these gains have come at the expense of seed protein content, which has declined across most breeding programs, while seed oil content has shown only modest improvements. Seed compositional traits, particularly those related to nutritional quality, have received insufficient attention relative to yield, and physiological traits remain the least documented category of genetic gain globally. The principal component analysis further confirmed a clear temporal shift in the genetic architecture of modern cultivars, with newer releases clustering around higher yield, oil content, and altered plant morphology. Most of the genetic gains reported in this review were achieved primarily through conventional phenotypic selection, whereas modern soybean breeding programs are becoming increasingly shaped by biotechnological and molecular tools. The advantages of approaches such as genotyping by sequencing (GBS), gene editing (e.g., CRISPR/Cas9), and genomic selection are that they enable precise, rapid identification of desirable alleles and shorten the lengthy phenotypic selection process. However, it is still important to integrate conventional breeding strategies with molecular breeding to enhance genetic improvement, especially in countries with limited breeding infrastructure. Critically, this review identifies the absence of phytic acid (PA) among tracked compositional traits in genetic gain studies as a significant gap in the literature. Given that PA is a chelator of iron (Fe) and other essential cation micronutrients, high PA concentration in soybean seeds fundamentally limits Fe bioavailability regardless of how much has been accumulated through breeding. Future breeding programs must, therefore, systematically incorporate PA alongside Fe as complementary targets and evaluate these traits under varying environmental production conditions/regions, as genotype-by-environment interactions can significantly alter the PA:Fe ratio. Developing soybean cultivars that are simultaneously high-yielding, high in bioavailable iron, and low in PA under differing water stress regimes remains a nutritionally urgent and scientifically justified breeding objective.

## Figures and Tables

**Table 1:** Genetic gains for yield and related agronomic and physiological attributes, and compositional traits in soybean.

Traits	Country	Year of Cultivar Release	Mean Annual Increase/Decrease	Annual Genetic Gain	References
*Agronomic traits*					
Days to flowering	Nigeria	1980–1996	−0.12 days year^−1^	−0.25% year^−1^	[14]
	China	1958–2001	−0.11 days year^−1^	-	[16]
	Nigeria	1980–1996	−0.059 days year^−1^	−0.11% year^−1^	[14]
	China	1958–2001	0.08 days year^−1^	-	[16]
	Zimbabwe	1966–2012	0.15 days year^−1^	0.32% year^−1^	[38]
	Zimbabwe	1966–2013	0.17 days year^−1^	0.35% year^−1^	[38]
Days to maturity	USA	1940–2000	−1 days year^−1^	-	[39]
	Brazil	2000–2017	−0.37 days year^−1^	−0.44% year^−1^	[29]
	Brazil	1965–2011	−0.29 days year^−1^	-	[17]
	Argentina	1980–2015	−0.17 days year^−1^	−0.12% year^−1^	[40]
	USA	1928–2008	0.022 days year^−1^	-	[41]
	USA	1928–2008	0.054 days year^−1^	-	[41]
	Nigeria	1980–1996	0.09 days year^−1^	0.08% year^−1^	[14]
	USA	1923–2008	0.09 days year^−1^	-	[13]
	USA	1923–2008	0.09 days year^−1^	-	[13]
	USA	1923–2008	0.10 days year^−1^	-	[13]
	Nigeria	1980–1996	0.11 days year^−1^	0.11% year^−1^	[14]
	USA	1923–2008	0.12 days year^−1^	-	[42]
	Zimbabwe	1966–2012	0.15 days year^−1^	0.27% year^−1^	[38]
	China	1995–2016	0.21 days year^−1^	4.00% year^−1^	[43]
	China	1995–2016	0.22 days year^−1^	4.00% year^−1^	[43]
	Argentina	1980–2015	0.23 days year^−1^	0.21% year^−1^	[40]
	Argentina	1980–2015	0.26 days year^−1^	0.16% year^−1^	[40]
	Zimbabwe	1966–2013	0.35 days year^−1^	0.32% year^−1^	[38]
	USA	1950–2000	1.00 days year^−1^	-	[39]
	USA	1920–2000	1.00 days year^−1^	-	[39]
	USA	-	-	0.49% year^−1^	[44]
	USA	-	-	0.3% year^−1^	[44]
	USA	-	-	−0.61% year^−1^	[44]
Plant height	USA	1920–2000	−2.1 cm year^−1^	-	[39]
	USA	1940–2000	−1.6 cm year^−1^	-	[39]
	Nigeria	1980–1996	−0.76 cm year^−1^	−0.98% year^−1^	[14]
	Brazil	2000–2017	−0.59 cm year^−1^	0.32% year^−1^	[29]
	Brazil	1965–2011	−0.56 cm year^−1^	-	[17]
	India	1969–2008	−0.37 cm year^−1^	-	[45]
	USA	1923–2008	−0.28 cm year^−1^	-	[46]
	USA	1923–2008	−0.21 cm year^−1^	-	[46]
	USA	1923–2008	−0.21 cm year^−1^	-	[13]
	USA	1923–2008	−0.18 cm year^−1^	-	[42]
	USA	1923–2008	−0.13 cm year^−1^	-	[13]
	USA	1923–2008	−0.13 cm year^−1^	-	[13]
	USA	1928–2008	−0.12 cm year^−1^	-	[41]
	USA	1928–2008	−0.06 cm year^−1^	-	[41]
	China	1929–2004	−0.01 cm year^−1^	-	[47]
	Zimbabwe	1966–2013	0.1 cm year^−1^	0.11% year^−1^	[38]
	Zimbabwe	1966–2012	0.37 cm year^−1^	0.50% year^−1^	[38]
	China	1958–2001	0.5 cm year^−1^	-	[16]
	Nigeria	1980–1996	0.63 cm year^−1^	1.66% year^−1^	[14]
	USA	1950–2000	8.7 cm year^−1^	-	[38]
	China	1950–2006	-	−13.8% year^−1^	[48]
Lodging	Zimbabwe	1966–2012	−0.33 units year^−1^	−0.02% year^−1^	[38]
	Zimbabwe	1966–2013	−0.31 units year^−1^	−2.08% year^−1^	[38]
	China	1929–2004	−0.09 units year^−1^	-	[47]
	Brazil	1965–2011	−0.07 units year^−1^	-	[17]
	China	1958–2001	−0.07 units year^−1^	-	[16]
	China	1958–2001	−0.062 units year^−1^	-	[16]
	India	1969–2008	−0.032 units year^−1^	-	[45]
	China	1958–2001	−0.03 units year^−1^	-	[16]
	Nigeria	1980–1996	−0.02 units year^−1^	−1.07% year^−1^	[14]
	USA	1923–2008	−0.02 units year^−1^	-	[42]
	USA	1923–2008	−0.018 units year^−1^	-	[13]
	USA	1923–2008	−0.014 units year^−1^	-	[13]
	USA	1928–2008	−0.013 units year^−1^	-	[41]
	USA	1923–2008	−0.012 units year^−1^	-	[13]
	Nigeria	1980–1996	−0.01 units year^−1^	−0.80% year^−1^	[14]
	USA	1923–2008	−1.8 units year^−1^	-	[49]
	USA	1923–2008	−2.4 units year^−1^	-	[49]
	USA	-	-	−0.08% year^−1^	[44]
	USA	-	-	−0.11% year^−1^	[44]
	China	1950–2006	-	−1.22% year^−1^	[48]
Number of branches	Brazil	1965–2011	−0.12 units year^−1^	-	[17]
	China	1958–2001	−0.028 units year^−1^	-	[16]
	China	1958–2001	0.01 units year^−1^	-	[16]
Number of pods per plants	Nigeria	1980–1996	−0.22 units year^−1^	−0.16% year^−1^	[14]
	China	1929–2004	−0.004 units year^−1^	-	[47]
	Brazil	2000–2017	0.11 units year^−1^	2.31% year^−1^	[29]
	India	1969–2008	0.281 units year^−1^	-	[45]
	Brazil	1965–2011	0.64 units year^−1^	1.51% year^−1^	[17]
	China	1995–2016	1.8 units year^−1^	-	[43]
	China	1995–2016	2.1 units year^−1^	-	[43]
	Nigeria	1980–1996	2.67 units year^−1^	3.51% year^−1^	[14]
	India	1969–1993		−4.00% year^−1^	[50]
Number of seeds per pod	Brazil	2000–2017	−0.45 units year^−1^	−1.10% year^−1^	[29]
	USA	1923–2008	0.002 units year^−1^	-	[46]
	USA	1923–2008	0.0022 units year^−1^	-	[46]
	China	1958–2001	0.004 units year^−1^	-	[16]
	China	1929–2004	0.005 units year^−1^	-	[47]
	Brazil	1965–2011	0.01 units year^−1^	-	[17]
	India	1969–2008	0.011 units year^−1^	-	[45]
	China	1995–2016	1.6 units year^−1^	-	[43]
	China	1995–2016	1.9 units year^−1^	-	[43]
	India	1969–1993	-	10.00% year^−1^	[50]
Number of seeds per plant	China	1929–2004	−0.31 units year^−1^	-	[47]
	China	1958–2001	0.25 units year^−1^	-	[16]
	China	1950–2006	-	0.41% year^−1^	[48]
	Canada	1934–1992	-	0.49% year^−1^	[51]
Total biomass	Nigeria	1980–1996	−6.07 g m^−2^ year^−1^	−0.41% year^−1^	[14]
	China	1995–2016	0.9 g m^−2^ year^−1^	-	[43]
	China	1995–2016	1.3 g m^−2^ year^−1^	-	[43]
	Nigeria	1980–1996	16.18 g m^−2^ year^−1^	1.31% year^−1^	[14]
	Brazil	1965–2011	81.17 g m^−2^ year^−1^	-	[17]
	China	1950–2006	-	0.19% year^−1^	[48]
	India	1969–1993	-	5.00% year^−1^	[50]
Hundred seed weight	USA	1923–2008	−1.14 g year^−1^	7.70% year^−1^	[15]
	USA	1923–2008	−0.019 g year^−1^	-	[13]
	China	1929–2004	0.002 g year^−1^	-	[47]
	USA	1923–2008	0.002 g year^−1^	-	[13]
	USA	1923–2008	0.01 g year^−1^	-	[13]
	USA	1928–2008	0.017 g year^−1^	-	[52]
	China	1958–2001	0.04 g year^−1^	-	[16]
	Brazil	1965–2011	0.055 g year^−1^	-	[17]
	Zimbabwe	1966–2012	0.09 g year^−1^	0.48% year^−1^	[38]
	Brazil	2000–2017	0.16 g year^−1^	2.69% year^−1^	[29]
	Zimbabwe	1966–2013	0.21 g year^−1^	1.16% year^−1^	[38]
	India	1969–2008	1.00 g year^−1^	-	[45]
	USA	1923–2008	1.02 g year^−1^	7.80% year^−1^	[15]
Seed yield	USA	1928–2008	3.1 kg ha^−1^ year^−1^	-	[52]
	Brazil	-	4.7 kg ha^−1^ year^−1^	-	[53]
	China	1958–2001	6 kg ha^−1^ year^−1^	0.63% year^−1^	[16]
	China	1929–2004	9.97 kg ha^−1^ year^−1^	0.60% year^−1^	[47]
	USA	1928–2008	10.3 kg ha^−1^ year^−1^	0.60% year^−1^	[41]
	Canada	1934–1994	12 kg ha^−1^ year^−1^	0.50% year^−1^	[54]
	USA	1920–2000	12.4 kg ha^−1^ year^−1^	37.90% year^−1^	[39]
	USA	1928–2008	13.5 kg ha^−1^ year^−1^	0.70% year^−1^	[41]
	USA	1928–2008	13.7 kg ha^−1^ year^−1^	0.70% year^−1^	[41]
	China	1958–2001	14 kg ha^−1^ year^−1^	0.56% year^−1^	[16]
	USA	1954–1987	14.4 kg ha^−1^ year^−1^	-	[22]
	China	1958–2001	16 kg ha^−1^ year^−1^	0.54% year^−1^	[16]
	USA	1940–2000	16.4 kg ha^−1^ year^−1^	38.10% year^−1^	[39]
	USA	1923–2008	17.2 kg ha^−1^ year^−1^	-	[15]
	Brazil	-	17.53 kg ha^−1^ year^−1^	-	[53]
	USA	1928–2008	17.6 kg ha^−1^ year^−1^	0.90% year^−1^	[41]
	USA	1972–2003	18.45 kg ha^−1^ year^−1^	1.05% year^−1^	[55]
	USA	1923–2008	19.3 kg ha^−1^ year^−1^	-	[46]
	USA	1923–2008	19.5 kg ha^−1^ year^−1^	-	[13]
	Brazil	2000–2017	20.8 kg ha^−1^ year^−1^	1.92% year^−1^	[29]
	USA	1923–2007	20.8 kg ha^−1^ year^−1^	-	[56]
	USA	1923–2008	20.9 kg ha^−1^ year^−1^	-	[49]
	USA	1950–2000	21.7 kg ha^−1^ year^−1^	58.50% year^−1^	[39]
	Nigeria	1980–1996	22.23 kg ha^−1^ year^−1^	1.42% year^−1^	[14]
	USA	1923–2008	22.8 kg ha^−1^ year^−1^	-	[13]
	USA	1923–2008	22.8 kg ha^−1^ year^−1^	-	[15]
	USA	1923–2008	22.8 kg ha^−1^ year^−1^	-	[42]
	India	1969–2008	23.08 kg ha^−1^ year^−1^	2.60% year^−1^	[45]
	USA	1923–2008	23.1 kg ha^−1^ year^−1^	-	[13]
	USA	1923–2008	23.4 kg ha^−1^ year^−1^	-	[49]
	Nigeria	1980–1996	23.61 kg ha^−1^ year^−1^	1.99% year^−1^	[14]
	USA	1923–2008	24.1 kg ha^−1^ year^−1^	-	[46]
	USA	1938–2004	25.4 kg ha^−1^ year^−1^	-	[34]
	USA	1972–2003	30.9 kg ha^−1^ year^−1^	1.20% year^−1^	[55]
	Brazil		31.22 kg ha^−1^ year^−1^	-	[53]
	USA	1923–2007	32.1 kg ha^−1^ year^−1^	-	[56]
	Brazil	1965–2011	40.06 kg ha^−1^ year^−1^	2.40% year^−1^	[17]
	Zimbabwe	1966–2012	41.02 kg ha^−1^ year^−1^	1.47% year^−1^	[38]
	Argentina	1980–2015	44.3 kg ha^−1^ year^−1^	1.10% year^−1^	[40]
	Zimbabwe	1966–2013	47.00 kg ha^−1^ year^−1^	1.67% year^−1^	[38]
	China	1995–2016	1.75–2.04 kg ha^−1^ year^−1^	-	[43]
	China	1995–2016	2.07–2.30 kg ha^−1^ year^−1^	-	[43]
	USA	-	-	−1.40% year^−1^	[44]
	USA	-	-	3.70% year^−1^	[44]
	USA	-	-	9.10% year^−1^	[44]
	China	1950–2006	-	0.58% year^−1^	[48]
	Canada	1934–1992	-	0.45% year^−1^	[51]
	Canada	1934–1992	-	0.50% year^−1^	[57]
	USA	1972–2003	-	1.20% year^−1^	[55]
	India	1969–1993	-	19.00% year^−1^	[50]
*Physiological traits*					
Stomatal conductance	Brazil	1965–2011	0.002 molH_2_O m^−2^ s^−1^ year^−1^	-	[17]
	Canada	1934–1992	-	0.48% year^−1^	[57]
Chlorophyll content a	Brazil	1965–2011	0.11 units year^−1^	-	[17]
Chlorophyll content b	Brazil	1965–2011	0.04 units year^−1^	-	[17]
Leaf area index	Brazil	1965–2011	-	0.31% year^−1^	[17]
	China	1950–2006	-	−0.31% year^−1^	[48]
	Canada	1934–1992	-	−0.38% year^−1^	[57]
Water use efficiency	Brazil	1965–2011	−0.002% year^−1^	-	[17]
Photosynthetic rate	Brazil	1965–2011	0.08 µmol CO_2_ m^−2^ s^−1^ year^−1^	-	[17]
	China	1950–2006	-	0.59% year^−1^	[48]
	Canada	1934–1992	-	0.52% year^−1^	[57]
Transpiration rate	Brazil	1965–2011	0.04 mmol m^−2^ s^−1^ year^−1^	-	[17]
*Compositional traits*					
Seed protein content	USA	1923–2008	−0.28 g kg^−1^ year^−1^	-	[49]
	USA	1923–2008	−0.22 g kg^−1^ year^−1^	-	[49]
	USA	1928–2008	−0.234 g kg^−1^ year^−1^	-	[41]
	USA	1923–2007	−0.242 g kg^−1^ year^−1^	-	[52]
	USA	1923–2008	−0.22 g kg^−1^ year^−1^	-	[13]
	USA	1923–2008	−0.22 g kg^−1^ year^−1^	-	[13]
	USA	1928–2008	−0.191 g kg^−1^ year^−1^	-	[52]
	USA	1923–2008	−0.159 g kg^−1^ year^−1^	-	[13]
	USA	1920–2000	−0.35 g kg^−1^ year^−1^	-	[39]
	USA	1940–2000	−0.32 g kg^−1^ year^−1^	-	[39]
	Zimbabwe	1966–2013	-	−0.01% year^−1^	[38]
	USA	1950–2000	0.18 g kg^−1^ year^−1^	-	[39]
	Canada	1934–1992	-	−0.13% year^−1^	[51]
	USA	1980–2013	-	−0.06% year^−1^	[58]
Seed oil content	Zimbabwe	1966–2013	-	0.004% year^−1^	[38]
	USA	1950–2000	0.062 g kg^−1^ year^−1^	-	[39]
	USA	1940–2000	0.15 g kg^−1^ year^−1^	-	[39]
	USA	1920–2000	0.26 g kg^−1^ year^−1^	-	[39]
	USA	1923–2008	0.051 g kg^−1^ year^−1^	-	[13]
	USA	1923–2008	0.103 g kg^−1^ year^−1^	-	[13]
	USA	1923–2007	0.127 g kg^−1^ year^−1^	-	[52]
	USA	1923–2008	0.136 g kg^−1^ year^−1^	-	[13]
	USA	1928–2008	0.142 g kg^−1^ year^−1^	-	[52]
	USA	1923–2008	0.11 g kg^−1^ year^−1^	-	[49]
	USA	1923–2008	0.13 g kg^−1^ year^−1^	-	[49]
	Canada	1934–1992	-	0.23% year^−1^	[51]

Where USA is the United States of America.

**Table 2:** The mean values, descriptive statistics, and test of significance for seed yield and related traits and for seed compositional attributes recorded globally from diverse soybean varieties and production regions.

Country	DTF	DTM	PH	LOD	NB	NPP	NSPod	NSP	SY	HSW	TB	SPC	SOC
Bangladesh	63.81	123.13	65.87	-	3.57	48.10	1.96	-	3220.81	11.91	-	-	-
Brazil	-	77.74	94.15	2.92	-	66.80	-	-	3400.31	15.60	-	-	-
Canada	-	-	-	-	-	-	-	-	-	16.53	-	-	-
China	-	118.24	-	-	-	-	-	-	2160.35	-	-	-	-
Colombia	36.48	85.66	70.51	-	-	-	-	-	-	12.10	-	-	-
Croatia		-	99.90	-	36.75	-	-	-	3719.46	-	-	38.00	20.25
Ethiopia	55.70	121.92	48.20	1.17	3.87	27.71	3.10	52.60	1657.24	-	9344.01	-	-
India	45.00	105.47	70.82	-	5.43	52.21	2.01	86.28	1710.34	14.01	6521.60	-	18.85
Nigeria	47.89	112.00	48.84	1.38	3.42	78.82	-	-	1753.33	-	1819.81	-	-
South Korea	-	118.11	-	-	-	-	-	-	-	-	-	-	-
USA	-	51.50	96.02	2.03	-	-	2.37	-	3125.33	-	-	42.15	20.95
Vietnam	38.45	104.22	32.50	-	-	-	-	-	-	17.31	-	-	-
Zimbabwe	52.67	124.76	87.40	-	-	-	-	-	4200.00	21.79	-	39.78	16.93
**Grand mean**	48.57	103.89	71.42	1.88	10.61	54.73	2.36	69.44	2771.91	15.61	5895.14	39.98	19.25
**SD**	9.68	23.17	23.06	0.79	14.64	19.40	0.53	23.82	12,369.67	3.43	3801.02	2.08	1.78
**Minimum**	36.48	51.50	32.50	1.17	3.42	27.71	1.96	52.60	1657.24	11.91	1819.81	38.00	16.93
**Maximum**	63.81	124.76	99.90	2.92	36.75	78.82	3.10	86.28	4200.00	21.79	9344.01	42.15	20.95
**Kurtosis**	−0.67	1.32	−1.09	−0.48	4.94	−0.13	1.36	-	8.85	0.74	-	-	−0.61
***p*-values**	*<0.001*	*<0.001*	*<0.001*	*-*	-	-	*<0.001*	-	*<0.001*	*n.s*	*<0.001*	*n.s*	*n.s*
**LSD**	2.06	2.899	9.79	-	-	-	-	-	211.1	14.44	132.7	-	-
**SED**	1.04	1.47	4.95	-	-	-	1.161	-	107.3	7.282	66.3	0.09	0.06

Where DTF—number of days to flowering (in days), DTM—number of days to maturity (in days), PH—plant height (in cm), LOD—lodging, NB—number of branches (number), NPP—number of pods per plant (number), NSPod—number of seeds per pod (number), NSP—number of seeds per plant (number), SY—seed yield (Kg/ha), HSW—hundred seed weight (g), TB—total biomass (Kg/ha), SPC—seed protein content (%), SOC—seed oil content (%), SD—standard deviation, LSD—least significant difference, SED—standard error of the difference, and n.s—not significant. Relationship between year of cultivar release and the performance of recorded yield and related traits, and compositional attributes in soybean.

**Table 3:** The Pearson correlation coefficients (*r*) for yield and related traits, and for compositional attributes recorded across decades and diverse global production regions in soybean.

Traits	SY	SPC	SOC	DTF	DTM	PH	NPP	NSPod	NSP	TB	HSW	LOD
**SY**												
**SPC**	−0.49 ***											
**SOC**	0.404 **	−0.609 ***										
**DTF**	0.504 ***	0.077	0.06									
**DTM**	0.101	0.005	0.166	0.829 ***								
**PH**	0.45 ***	−0.018	0.164	0.548 ***	0.044							
**NPP**	0.021	-	-	0.58 ***	0.378 ***	0.067						
**NSPod**	0.841 ***	-	-	−0.391 **	−0.912 ***	0.309 *	0.443 ***					
**NSP**	0.657 **	-	-	0.053	-	−0.514 *	0.897 ***	-				
**TB**	0.263 *	-	-	−0.466 ***	−0.441 ***	0.467 ***	−0.838 ***	0.084	-			
**HSW**	0.255 **	−0.431 **	0.311 *	0.062	0.132	−0.011	−0.06	0.006	0.266	−0.09		
**LOD**	−0.331	-	-	0.234	−0.038	0.557 **	−0.114	-	-	0.041	-	

Where DTF—number of days to flower (in days), DTM—number of days to maturity (in days), PH—plant height (in cm), LOD—lodging, NPP—number of pods per plant (number), NSPod—number of seeds per pod (number), NSP—number of seeds per plant (number), SY—seed yield (Kg/ha), HSW—hundred seed weight (g), TB—total biomass (Kg/ha), SPC—seed protein content (%), SOC—seed oil content (%), * significant (*p* < 0.05), ** highly significant (*p* < 0.01), and *** highly significant (*p* < 0.001). Principal component biplot analysis for the agronomic and biochemical traits recorded across decades of cultivar release and production regions.

**Table 4:** The selected studies used to compile databases of annual genetic gain for yield and related traits, and for compositional attributes in soybean.

Country Name	Cultivar Year of Release	Author
Argentina	1980–2015	[40]
USA	1928–2008	[52]
USA	1923–2007	[52]
USA	1923–2008	[42]
USA	1923–2008	[15]
USA	1923–2008	[49]
USA		[44]
Brazil		[53]
USA	1928–2008	[41]
Brazil	1965–2011	[17]
USA	1923–2008	[13]
China	1929–2004	[47]
USA	1923–2007	[56]
Canada	1934–1994	[54]
China	1950–2006	[48]
Canada	1934–1992	[51]
Nigeria	1980–1996	[14]
USA	1980–2013	[58]
China	1995–2016	[43]
USA	1923–2008	[46]
USA	1938–2004	[34]
USA	1950–2000	[39]
USA	1940–2000	[39]
USA	1920–2000	[39]
Brazil	2000–2017	[29]
Zimbabwe	1966–2013	[38]
Zimbabwe	1966–2012	[38]
USA	1972–2003	[55]
USA	1954–1987	[22]
India	1969–2008	[45]
China	1958–2001	[16]
India	1969–1993	[50]

Where USA is the United States of America.

**Table 5:** Description of agronomic, physiological, and biochemical traits reviewed in the study.

Traits	Symbols	Units	Definition
*Agronomic traits*			
Number of days to flowering	DTF	days	The number of days from sowing to 50% of the plants producing flowers
Number of days to maturity	DTM	days	The number of days from sowing to 50% of the plants showing dry and brown coloured pods
Plant height	PH	cm	The height from soil surface or base of the plant to the longest trifoliate leaf
Lodging	LOD		The permanent bending or displacement from its vertical position of the plant’s stem
Number of branches	NB		The total number of branches produced by the plants
Number of pods per plant	NPP		The total number of pods produced by a single plant
Number of seeds per pod	NSPod		The total number of seeds produced by a single pod
Number of seeds per plant	NSP		The total number of seeds produced by a single plant
Total biomass	TB	Kg ha^−1^	The weight of the below- and above-ground biomass
Hundred seeds weight	HSW	g	The total weight of 100 individual seeds
Seed yield	SY	Kg ha^−1^	The total weight of the seeds produced by the plant
*Physiological traits*			
Stomatal conductance		molH_2_O m^−2^ s^−1^	The rate of gas exchange through the stomata of the leaf
Chlorophyll content (a and b)			The amount of chlorophyll a and b found in the plant
Leaf area index	LAI		The ratio of the area of the leaf to the area of the plot
Water use efficiency	WUE	%	The ratio of the yield produced to the amount of water applied in the plot
Photosynthetic rate		µmolCO_2_ m^−2^ s^−1^	The rate at which the plant produces oxygen per plant tissue area
Transpiration rate		mmol m^−2^ s^−1^	The rate at which the plant releases water to the atmosphere per area
*Biochemical traits*			
Seed protein content	SPC	%	The total amount of protein produced by the seeds of the plant
Seed oil content	SOC	%	The total amount of oil produced by the seeds of the plant

## Data Availability

The data presented in this study are available in the article and Supplementary Materials. The dataset was compiled from the published studies cited within this manuscript.

## Supplementary Materials

The following supporting information can be downloaded at https://www.mdpi.com/article/10.3390/plants15172677/s1, Table S1. The selected studies included in the database of annual mean changes for yield and related traits, and for compositional attributes in soybean [78,79,80,81,82,83,84,85,86,87,88,89,90,91,92,93,94].
