# Supplementary material for: Global Genetic Gain and Changes for Seed Yield, Agronomic, and Compositional Attributes in Soybean (Glycine max [L.] Merr.) from the 1920s to the 2010s: A Meta-Analysis
Source: Plants (Basel). 2026 Aug 31;15(17):2677. doi: 10.3390/plants15172677 (PMC13567543; doi:10.3390/plants15172677)
Supplement: Supplementary file 1 [file plants-15-02677-s001.zip › plants-4447407-supplementary.pdf]

Table S1. The selected studies included in the database of annual mean changes for yield and related traits, and for compositional attributes in soybean.

Country name	Cultivar name	Cultivar year of release	Author
USA	-	-	[78]
Ethopia	-	-	[79]
USA	-	1928-2008	[41]
USA	-	-	[80]
India	-	-	[81]
USA	-	-	[82]
USA	-	1997-2014	[83]
Brazil	-	-	[84]
Brazil	-	-	[53]
Canada	Pagoda	1939	[54]
Canada	Maple Glen	1987	[54]
Canada	Mandarin	1934	[54]
Canada	OAC Bayfield	1994	[54]
USA	Graham	1996	[54]
USA	Anand	1999	[54]
USA	Pace	1996	[54]
USA	Musen	1997	[54]
USA	TN-5-95	1997	[54]
USA	Clifford	1994	[54]
USA	Ransom	1970	[54]
USA	Bragg	1963	[54]
USA	Mack	1971	[54]
USA	Lyon	1993	[54]
USA	Hutton	1972	[54]
USA	Essex	1972	[54]

Country name	Cultivar name	Cultivar year of release	Author
USA	Jackson	1953	[54]
USA	Semmes	1965	[54]
USA	Lee	1954	[54]
USA	Tracy	1973	[54]
USA	Dyer	1967	[54]
USA	Hardee	1962	[54]
Nigeria	Samsoy-1	1980	[14]
Nigeria	TGx 1019-2EN	1980	[14]
Nigeria	TGx 1805-31F	1986	[14]
Nigeria	TGx 1838-5E	1986	[14]
Nigeria	TGx 1869-31E	1991	[14]
Nigeria	TGx 1894-3F	1991	[14]
Nigeria	TGx 1880-3E	1991	[14]
Nigeria	TGx 1888-15F	1991	[14]
Nigeria	TGx 1903-5F	1996	[14]
Nigeria	TGx 1910-7F	1996	[14]
Nigeria	Samsoy-2	1980	[14]
Nigeria	TGx 923-2E	1980	[14]
Nigeria	TGx 1440-1E	1986	[14]
Nigeria	TGx 1448-2E	1986	[14]
Nigeria	TGx 1844-4E	1991	[14]
Nigeria	TGx 1844-18E	1991	[14]
Nigeria	TGx 1866-12F	1991	[14]
Nigeria	TGx 1889-12F	1991	[14]
Nigeria	TGx 1910-5F	1996	[14]
Nigeria	TGx 1910-6F	1996	[14]
Nigeria	TGx 1910-14F	1996	[14]
China	-	1995-2016	[43]
Croatia	-	-	[85]

Country name	Cultivar name	Cultivar year of release	Author
Vietnam	-	-	[86]
South Korea	-	-	[87]
Nigeria	AgbonKagoro	-	[88]
Nigeria	Akwanga	-	[88]
Nigeria	Andaha	-	[88]
Nigeria	Ashuku	-	[88]
Nigeria	Dadinkowa	-	[88]
Nigeria	Garkawa	-	[88]
Nigeria	Gwantu	-	[88]
Nigeria	Kafanchan	-	[88]
Nigeria	Kagoro	-	[88]
Nigeria	Langtang	-	[88]
Nigeria	Lau	-	[88]
Nigeria	Mangu	-	[88]
Nigeria	Mararaba	-	[88]
Nigeria	TGX1485-ID	-	[88]
Nigeria	TGX1448-2E	-	[88]
Nigeria	TGX1987-10F	-	[88]
Nigeria	TGX1835-10e	-	[88]
Nigeria	TGX1987-62F	-	[88]
Nigeria	Tiv Local	-	[88]
Nigeria	Vom	-	[88]
Colombia		-	[89]
USA		-	[90]
USA	Vinton 81	-	[91]
USA	Century 64	-	[91]
USA	HS89·2958	-	[91]
USA	HS89-2987	-	[91]
USA	Resnik	-	[91]

Country name	Cultivar name	Cultivar year of release	Author
USA	Edison	-	[91]
USA	Williams 82	-	[91]
USA	Keller	-	[91]
USA	Conrad	-	[91]
USA	Kenwood	-	[91]
USA	Sandusky .	-	[91]
USA	HS84-6247	-	[91]
Ethopia	T1-EL-OS-JM17-A13	-	[92]
Ethopia	T1-EL-OS-JM17-A15	-	[92]
Ethopia	T1-EL-OS-JM17-B11	-	[92]
Ethopia	T1-EL-OS-JM17-B14	-	[92]
Ethopia	T1-EL-OS-JM17-C2	-	[92]
Ethopia	T1-EL-OS-JM17-D4	-	[92]
Ethopia	T1-EL-OS-JM17-D6	-	[92]
Ethopia	T1-EL-OS-JM17-D14	-	[92]
Ethopia	T1-EL-OS-JM17-E1	-	[92]
Ethopia	T1-EL-OS-JM17-E2	-	[92]
Ethopia	T1-EL-OS-JM17-E3	-	[92]
Ethopia	T1-EL-OS-JM17-E5	-	[92]
Ethopia	T1-EL-OS-JM17-E6	-	[92]
Ethopia	T1-EL-OS-JM17-E13	-	[92]
Ethopia	T1-EL-OS-JM17-E15	-	[92]
Ethopia	T1-EL-OS-JM17-E18	-	[92]
Ethopia	T1-EL-OS-JM17-E23	-	[92]
Ethopia	T1-EL-OS-JM17-E27	-	[92]
Ethopia	T1-EL-OS-JM17-E28	-	[92]
Ethopia	T1-EL-OS-JM17-G13	-	[92]
Ethopia	T1-EL-OS-JM17-H6	-	[92]
Ethopia	T1-EL-OS-JM17-H9	-	[92]

Country name	Cultivar name	Cultivar year of release	Author
Ethopia	T1-EL-OS-JM17-H12	-	[92]
Ethopia	T2-EL-LG-90-JM17-I12	-	[92]
Ethopia	T2-EL-LG-90-JM17-I15	-	[92]
Ethopia	T2-EL-LG-90-JM17-I22	-	[92]
Ethopia	T3-EL-LG-63-JM17-A1	-	[92]
Ethopia	T3-EL-LG-63-JM17-A3	-	[92]
Ethopia	T3-EL-LG-63-JM17-A8	-	[92]
Ethopia	T3-EL-LG-63-JM17-A10	-	[92]
Ethopia	T3-EL-LG-63-JM17-A17	-	[92]
Ethopia	T3-EL-LG-63-JM17-A22	-	[92]
Ethopia	T3-EL-LG-63-JM17-A28	-	[92]
Ethopia	T3-EL-LG-63-JM17-B4	-	[92]
Ethopia	T3-EL-LG-63-JM17-B6	-	[92]
Ethopia	T3-EL-LG-63-JM17-B1	-	[92]
Ethopia	T3-EL-LG-63-JM17-C1	-	[92]
Ethopia	T3-EL-LG-63-JM17-E8	-	[92]
Ethopia	T3-EL-LG-63-JM17-E14	-	[92]
Ethopia	T3-EL-LG-63-JM17-E17	-	[92]
Ethopia	T3-EL-LG-63-JM17-E30	-	[92]
Ethopia	T3-EL-LG-63-JM17-E31	-	[92]
Ethopia	T3-EL-LG-63-JM17-FI1	-	[92]
Ethopia	T3-EL-LG-63-JM17-F29	-	[92]
Ethopia	T3-EL-LG-63-JM17-I31	-	[92]
Ethopia	T3-EL-LG-63-JM17-I36	-	[92]
Ethopia	T4-EL-LG-65-JM17-A8	-	[92]
Ethopia	T4-EL-LG-65-JM17-A11	-	[92]
Ethopia	T4-EL-LG-65-JM17-A13	-	[92]
Ethopia	T4-EL-LG-65-JM17-B8	-	[92]
Ethopia	T4-EL-LG-65-JM17-C15	-	[92]

Country name	Cultivar name	Cultivar year of release	Author
Ethopia	T4-EL-LG-65-JM17-C18	-	[92]
Ethopia	T4-EL-LG-65-JM17-C20	-	[92]
Ethopia	T4-EL-LG-65-JM17-E24	-	[92]
Ethopia	T4-EL-LG-65-JM17-F2	-	[92]
Ethopia	T4-EL-LG-65-JM17-G1	-	[92]
Ethopia	T4-EL-LG-65-JM17-G9	-	[92]
Ethopia	T4-EL-LG-65-JM17-G27	-	[92]
Ethopia	T4-EL-LG-65-JM17-I14	-	[92]
Ethopia	T5-EL-LD-77-JM17-A2B	-	[92]
Ethopia	T5-EL-LD-77-JM17-A5	-	[92]
Ethopia	T5-EL-LD-77-JM17-A7	-	[92]
Ethopia	T5-EL-LD-77-JM17-A9	-	[92]
Ethopia	T5-EL-LD-77-JM17-A11	-	[92]
Ethopia	T5-EL-LD-77-JM17-A14	-	[92]
Ethopia	T5-EL-LD-77-JM17-A15	-	[92]
Ethopia	T5-EL-LD-77-JM17-A17	-	[92]
Ethopia	T5-EL-LD-77-JM17-B16	-	[92]
Ethopia	T5-EL-LD-77-JM17-C1	-	[92]
Ethopia	T5-EL-LD-77-JM17-C3	-	[92]
Ethopia	T5-EL-LD-77-JM17-C4	-	[92]
Ethopia	T5-EL-LD-77-JM17-C6	-	[92]
Ethopia	T5-EL-LD-77-JM17-C25	-	[92]
Ethopia	T5-EL-LD-77-JM17-D4	-	[92]
Ethopia	T5-EL-LD-77-JM17-E10	-	[92]
Ethopia	T5-EL-LD-77-JM17-E25	-	[92]
Ethopia	T5-EL-LD-77-JM17-C25	-	[92]
Ethopia	T5-EL-LD-77-JM17-D4	-	[92]
Ethopia	T5-EL-LD-77-JM17-E10	-	[92]
Ethopia	T5-EL-LD-77-JM17-E25	-	[92]

Country name	Cultivar name	Cultivar year of release	Author
Ethopia	T5-EL-LD-77-JM17-E27	-	[92]
Ethopia	T5-EL-LD-77-JM17-F3	-	[92]
Ethopia	T5-EL-LD-77-JM17-F4	-	[92]
Ethopia	T5-EL-LD-77-JM17-F11	-	[92]
Ethopia	T5-EL-LD-77-JM17-F15	-	[92]
Ethopia	T5-EL-LD-77-JM17-F27	-	[92]
Ethopia	T5-EL-LD-77-JM17-F28	-	[92]
Ethopia	T5-EL-LD-77-JM17-G7	-	[92]
Ethopia	T5-EL-LD-77-JM17-G18	-	[92]
Ethopia	T5-EL-LD-77-JM17-G29	-	[92]
Ethopia	T5-EL-LD-77-JM17-H1	-	[92]
Ethopia	T5-EL-LD-77-JM17-H3	-	[92]
Ethopia	T5-EL-LD-77-JM17-H30	-	[92]
Ethopia	T5-EL-LD-77-JM17-H31	-	[92]
Ethopia	T5-EL-LD-77-JM17-H43	-	[92]
Ethopia	T5-EL-LD-77-JM17-H44	-	[92]
Ethopia	T5-EL-LD-77-JM17-I3	-	[92]
Ethopia	T5-EL-LD-77-JM17-I22	-	[92]
Ethopia	T5-EL-LD-77-JM17-I24	-	[92]
Ethopia	T5-EL-LD-77-JM17-I34	-	[92]
Ethopia	T5-EL-LD-77-JM17-I35	-	[92]
Ethopia	Nyala	-	[92]
Ethopia	Clarck 63K	-	[92]
Bangladesh	SBM-01	-	[93]
Bangladesh	SBM-02	-	[93]
Bangladesh	SBM-03	-	[93]
Bangladesh	SBM-04	-	[93]
Bangladesh	SBM-05	-	[93]
Bangladesh	SBM-06	-	[93]

Country name	Cultivar name	Cultivar year of release	Author
Bangladesh	SBM-08	-	[93]
Bangladesh	SBM-09	-	[93]
Bangladesh	SBM-10	-	[93]
Bangladesh	SBM-24	-	[93]
Bangladesh	SBM-25	-	[93]
Bangladesh	SBM-26	-	[93]
Bangladesh	Sohag	-	[93]
Bangladesh	SBM-11	-	[93]
Bangladesh	SBM-12	-	[93]
Bangladesh	SBM-13	-	[93]
Bangladesh	SBM-14	-	[93]
Bangladesh	BDS-4	-	[93]
Bangladesh	SBM-15	-	[93]
Bangladesh	SBM-16	-	[93]
Bangladesh	SBM-17	-	[93]
Bangladesh	SBM-18	-	[93]
Bangladesh	SBM-19	-	[93]
Bangladesh	SBM-20	-	[93]
Bangladesh	SBM-21	-	[93]
Bangladesh	SBM-22	-	[93]
Bangladesh	SBM-23	-	[93]
Bangladesh	BARI-5	-	[93]
Bangladesh	SBM-27	-	[93]
Bangladesh	SBM-28	-	[93]
Bangladesh	BAU-S/64	-	[93]
USA	Hardin	1983	[34]
USA	Harosoy	1951	[34]
USA	Hawkeye	1948	[34]
USA	Lincoln	1944	[34]

Country name	Cultivar name	Cultivar year of release	Author
USA	Richland	1938	[34]
USA	Williams 82	1981	[34]
USA	2509CN (PI 88788)	2003	[34]
USA	Ag2801 (PI 88788)	2003	[34]
USA	Dwight (PI 88788)	1997	[34]
USA	E2620RX (PI 437654	2003	[34]
USA	IA2068 (PI 88788)	2003	[34]
USA	L2811RX (PI 437654)	2003	[34]
USA	P91M90 (Peking)	2003	[34]
USA	PB291N (PI 88788)	2003	[34]
USA	S-3012-4 (PI 88788)	2004	[34]
USA	SOI2642NRR (PI 88788)	2003	[34]
USA	SOI2858NRR (PI 88788)	2003	[34]
USA	Ag2403	2004	[34]
USA	NE3001	2001	[34]
USA	NK S32-G5	2003	[34]
USA	P92M91	2004	[34]
USA	S25J5	2003	[34]
USA	S-2743-4	2004	[34]
USA	Harosoy	-	[34]
USA	Richland	-	[34]
USA	NK S32-G5	-	[34]
USA	S25J5	-	[34]
USA	PB291N	-	[34]
USA	SOI2642NRR	-	[34]
China	Xiaojinhuang	1951	[94]
China	Zihua 4 hao	1952	[94]
China	Yuanbaojin	1953	[94]
China	Ke 4430-20	1959	[94]

Country name	Cultivar name	Cultivar year of release	Author
China	Fengshou6hao	1960	[94]
China	Hejiao 6 hao	1962	[94]
China	Keshansilijia	1962	[94]
China	Dongnong 4 hao	1963	[94]
China	Fengshou 10	1966	[94]
China	Xiaolidou 9 hao	1967	[94]
China	Heinong10	1969	[94]
China	Heinong16	1970	[94]
China	Mufeng 5 hao	1972	[94]
China	Nenfeng 1 hao	1972	[94]
China	Suinong 3 hao	1973	[94]
China	Hefeng 22	1974	[94]
China	Nenfeng 4 hao	1975	[94]
China	Hefeng 23	1977	[94]
China	Fengshou 17	1977	[94]
China	Hongfeng 2 hao	1977	[94]
China	Nenfeng 9 hao	1980	[94]
China	Suinong 4 hao	1981	[94]
China	Heinong 27	1983	[94]
China	Suinong 5 hao	1984	[94]
China	Dongnong 37	1984	[94]
China	Beifeng 3 hao	1984	[94]
China	Hefeng 25	1984	[94]
China	Beifeng 5 hao	1986	[94]
China	Suinong 8 hao	1989	[94]
China	Heinong 35	1990	[94]
China	Suinong 9 hao	1991	[94]
China	Kenong 4 hao	1992	[94]
China	Suinong 10	1994	[94]

Country name	Cultivar name	Cultivar year of release	Author
China	Suinong 11	1995	[94]
China	Beifeng 11	1995	[94]
China	Suinong 12	1996	[94]
China	Suinong 14	1996	[94]
China	Suinong 15	1998	[94]
China	Heinong 45	2003	[94]
China	Kenfeng 12	2004	[94]
China	Suinong 21	2004	[94]
China	Hefeng 47	2004	[94]
China	Suinong 22	2005	[94]
China	Hai 635	2006	[94]
China	Hai 5052	2006	[94]
USA	5002T	2002	[39]
USA	R01-4804	2005	[39]
USA	Narrow	1985	[39]
USA	Ozark	2003	[39]
USA	UA-4805	2006	[39]
USA	CAVINESS	1988	[39]
USA	MD-92-576	2005	[39]
USA	R95-1705	2008	[39]
USA	Essex	1973	[39]
USA	R02-3263	2005	[39]
USA	AG4403	2003	[39]
USA	Manokin	2006	[39]
USA	Dare	1966	[39]
USA	Hill	1959	[39]
USA	Dorman	1952	[39]
USA	R97-1634	2005	[39]
USA	5601T	2001	[39]

Country name	Cultivar name	Cultivar year of release	Author
USA	UARK5896	2000	[39]
USA	AG5501	2001	[39]
USA	R01-4834	2005	[39]
USA	R01-4752	2005	[39]
USA	Hutcheson	1988	[39]
USA	Walters	1990	[39]
USA	Caviness-RR	2004	[39]
USA	DP5634RR	2003	[39]
USA	UARK5798	1999	[39]
USA	Forrest	1973	[39]
USA	Bedford	1978	[39]
USA	Hood	1960	[39]
USA	Ogden	1942	[39]
USA	NC-ROY	2001	[39]
USA	R98-209	2005	[39]
USA	Desha	2003	[39]
USA	Lonoke	2003	[39]
USA	Dillon	1997	[39]
USA	BOGGSRR	2000	[39]
USA	Young	1987	[39]
USA	Lee	1958	[39]
USA	Hood75	1975	[39]
USA	Pickett71	1971	[39]
USA	Pickett	1966	[39]
USA	Centennial	1977	[39]
USA	Lee68	1968	[39]
USA	Lee74	1974	[39]
USA	Arksoy	1928	[39]
Brazil		2000	[29]

Country name	Cultivar name	Cultivar year of release	Author
Brazil		2003	[29]
Brazil		2005	[29]
Brazil		2007	[29]
Brazil		2008	[29]
Brazil		2009	[29]
Brazil		2010	[29]
Brazil		2011	[29]
Brazil		2012	[29]
Brazil		2013	[29]
Brazil		2014	[29]
Brazil		2015	[29]
Brazil		2016	[29]
Brazil		2017	[29]
Zimbabwe	G16	2005	[38]
Zimbabwe	G15	2006	[38]
Zimbabwe	G17	2005	[38]
Zimbabwe	G1	2008	[38]
Zimbabwe	G5	2013	[38]
Zimbabwe	G21	2000	[38]
Zimbabwe	G28	1989	[38]
Zimbabwe	G14	2007	[38]
Zimbabwe	G25	1998	[38]
Zimbabwe	G42	2012	[38]
Zimbabwe	G7	2012	[38]
Zimbabwe	G40	1972	[38]
Zimbabwe	G19	2001	[38]
Zimbabwe	G8	2012	[38]
Zimbabwe	G20	2000	[38]
Zimbabwe	G6	1997	[38]

Country name	Cultivar name	Cultivar year of release	Author
Zimbabwe	G18	2003	[38]
Zimbabwe	G4	2005	[38]
Zimbabwe	G32	1988	[38]
Zimbabwe	G11	2008	[38]
Zimbabwe	G10	2008	[38]
Zimbabwe	G23	1999	[38]
Zimbabwe	G22	1999	[38]
Zimbabwe	G13	2007	[38]
Zimbabwe	G26	1997	[38]
Zimbabwe	G12	2007	[38]
Zimbabwe	G29	1995	[38]
Zimbabwe	G31	1992	[38]
Zimbabwe	G24	1999	[38]
Zimbabwe	G27	1994	[38]
Zimbabwe	G35	1980	[38]
Zimbabwe	G9	2010	[38]
Zimbabwe	G30	1992	[38]
Zimbabwe	G34	1982	[38]
Zimbabwe	G2	2008	[38]
Zimbabwe	G33	1985	[38]
Zimbabwe	G36	1977	[38]
Zimbabwe	G3	1999	[38]
Zimbabwe	G37	1977	[38]
Zimbabwe	G38	1974	[38]
Zimbabwe	G39	1973	[38]
Zimbabwe	G41	1966	[38]
USA		1972–1976	[55]
USA		1972–1976	[55]
USA		1972–1976	[55]

Country name	Cultivar name	Cultivar year of release	Author
USA		1999–2003	[55]
USA		1999–2003	[55]
USA		1999–2003	[55]
India	Kalitur	1969	[45]
India	Bragg	1969	[45]
India	Improved Pelican	1969	[45]
India	Hardee	1976	[45]
India	Punjab-1	1978	[45]
India	Durga	1981	[45]
India	Pusa 40	1981	[45]
India	PK308	1984	[45]
India	PK471	1988	[45]
India	JS 71-05	1990	[45]
India	JS335	1993	[45]
India	NRC 2	1997	[45]
India	PS 1042	1997	[45]
India	NRC 37	2001	[45]
India	JS 93-05	2002	[45]
India	JS 95-60	2006	[45]
India	PS 1347	2008	[45]
India	Bragg	1969	[50]
India	Lee	1969	[50]
India	Improved Pelican	1969	[50]
India	Hardee	1976	[50]
India	Monetta	1985	[50]
India	T 49	1978	[50]
India	Punjab I	1978	[50]
India	Shilajeet	1979	[50]
India	co 1	1980	[50]

Country name	Cultivar name	Cultivar year of release	Author
India	JS2	1982	[50]
India	Gujarat Soy I	1983	[50]
India	Gujarat Soy 2	1983	[50]
India	VL soy 2	1989	[50]
India	JS 71-05	1990	[50]
India	Ankur	1974	[50]
India	Alankar	1977	[50]
India	KHSb2	1979	[50]
India	Gaurav	1980	[50]
India	Durga	1981	[50]
India	Birsa Soy I	1981	[50]
India	Pusa 40	1981	[50]
India	VL soy 1	1982	[50]
India	PK 262	1982	[50]
India	PK 327	1982	[50]
India	Pusa 22	1983	[50]
India	JS 75-46	1984	[50]
India	MACS 13	1984	[50]
India	PK 308	1984	[50]
India	PK 416	1985	[50]
India	Pusa 37	1985	[50]
India	PK 472	1986	[50]
India	Shivalik	1987	[50]
India	Pusa 16	1987	[50]
India	Pusa 24	1987	[50]
India	Pusa 20	1988	[50]
India	PK 471	1988	[50]
India	PK 564	1988	[50]
India	MACS 58	1989	[50]

Country name	Cultivar name	Cultivar year of release	Author
India	JS 80-21	1990	[50]
India	MACS 124	1992	[50]
India	MACS 57	1992	[50]
India	JS 335	1993	[50]
India	NRC 2	1993	[50]

Where, the United State of America- USA
